# High-pressure processing reshapes early lipid mobilization in *Camellia oleifera* seeds during a hot–humid postharvest window

**DOI:** 10.3389/fpls.2026.1829285

**Published:** 2026-05-21

**Authors:** Jianwen Wu, Xingyue Tang, Suhua Yang, Jihua Guan, Guiqing Li, Mi Qiu

**Affiliations:** Guangxi Forestry Research Institute, Guangxi Laboratory of Forestry, Nanning, China

**Keywords:** *Camellia oleifera*, free fatty acids, high-pressure processing, lipid mobilization, lipidomics, postharvest

## Abstract

**Introduction:**

Freshly harvested *Camellia oleifera* (*C. oleifera*) fruits may experience a short hot–humid interval before routine drying, yet it remains unclear whether high-pressure processing (HPP) redirects seed lipid mobilization during this early postharvest window.

**Methods:**

We established a controlled seed-focused postharvest model in which fruits were subjected to HPP, and pericarps were removed before hot–humid holding to isolate seed-level responses rather than simulate whole-fruit commercial storage. Untargeted UHPLC–Orbitrap metabolomics was integrated with targeted GC–MS quantification of 49 free fatty acids (FFAs) across four regimes: untreated baseline, hot–humid holding alone (35 °C, 95% RH, 12 h), and the same holding step preceded by HPP at 100 or 500 MPa for 5 min.

**Results:**

Forty-two FFAs were detected with high within-regime reproducibility. Hot–humid holding alone caused only modest changes in total FFA abundance, whereas prior HPP converted the same holding period into a pressure-dependent lipid-mobilization response. The 500 MPa regime produced the strongest phenotype, characterized by a pronounced increase in total FFA burden and an unsaturation-enriched C18 free-acyl pool. Untargeted metabolomics further showed that this regime generated the broadest metabolic divergence, although its correspondence with targeted lipid metrics was selective rather than global. Semi-quantitative lipid summaries were compatible with membrane-lipid remodeling and oxygenation-associated metabolism as candidate contributors, but did not resolve the dominant biochemical route.

**Discussion:**

These findings define a pressure-primed lipid-mobilization window in *C. oleifera* seeds and identify treatment sequence as a key determinant of early seed lipid trajectories before drying. Further validation under whole-fruit storage, drying, oil-quality assessment, and scale-up conditions is required before this seed-level model can be extended to broader postharvest management.

## 1 Introduction

*Camellia oleifera* Abel (*C. oleifera*) is a perennial woody oilseed crop in the Theaceae family and one of the major sources of edible vegetable oil in China. Its commercial value derives not only from high oil yield, but also from a favorable fatty-acid composition, in which unsaturated fatty acids generally account for more than 85% of total fatty acids and oleic acid is the dominant component (Chen et al., 2024; Xiong et al., 2025). In addition to bulk triacylglycerols, camellia oil contains minor bioactive constituents such as squalene, phytosterols, polyphenols, and fat-soluble vitamins, all of which contribute to its nutritional value and product differentiation in premium edible and cosmetic markets (Li et al., 2022). However, translating this compositional advantage into stable industrial-quality oil depends strongly on postharvest handling. Freshly harvested camellia seeds usually contain high moisture (~40–50%), creating a narrow front-end processing window in which delayed or suboptimal handling can accelerate biochemical deterioration and complicate downstream drying and extraction (Li et al., 2022). This early postharvest interval is therefore not merely logistical; it is a quality-sensitive transition phase during which lipid hydrolysis and broader metabolic reorganization may already begin before routine drying.

Recent studies have expanded the front-end pretreatment options for *C. oleifera* seed processing, shifting the focus from drying acceleration alone to the balance among tissue disruption, oil release, and lipid-quality preservation. Cold-plasma pretreatment has been reported to modify camellia-seed structure and improve oil yield, whereas thermal and thermal-assisted approaches, including microwave heating, infrared radiation, roasting, frying, infrared-assisted hot-air drying, and ultrasound–microwave drying, can influence drying behavior, matrix organization, phenolic retention, antioxidant capacity, storage stability, flavor formation, and lipid profiles in camellia-seed oil production (Chen et al., 2024; Li et al., 2025; Luo et al., 2025; Wang et al., 2022; Wu et al., 2025; Xiong et al., 2025). These advances share a common processing rationale: controlled physical perturbation may improve mass transfer or oil recovery, but the same perturbation may also alter hydrolytic and oxidative pathways that determine final oil quality. This trade-off is particularly important for oil-rich seeds, because pretreatments of camellia seeds have been shown to affect acid value and peroxide value, two routine indicators associated with hydrolytic and oxidative deterioration of edible oils (Wang et al., 2022).

Within this broader physical-pretreatment landscape, high-pressure processing (HPP) offers a mechanistically distinct strategy because it applies a largely nonthermal, isostatic stress that can affect cell integrity, membrane permeability, enzyme accessibility, and food-matrix organization (Huang et al., 2017; Janowicz and Lenart, 2018; Knorr et al., 2011). Industrially relevant HPP is generally discussed in the several-hundred-megapascal range, with short treatments at 500–600 MPa commonly considered for microbial inactivation, whereas lower-pressure exposure is better interpreted as a milder structural perturbation rather than a conventional inactivation regime (EFSA BIOHAZ Panel et al., 2022). However, technological feasibility in food HPP does not necessarily imply lipid-quality suitability in moist, oil-rich seed matrices. High pressure may modify tissue compartmentation, oxygen exposure, and enzyme–substrate contact, and HPP-associated lipid oxidation has been reported in susceptible fat-containing foods, especially when unsaturated lipids and endogenous pro-oxidant systems are present (Medina-Meza et al., 2014). For freshly harvested *C. oleifera* seeds, which retain high moisture before routine drying, these considerations raise a specific postharvest question: whether HPP simply facilitates subsequent processing or instead redirects the short hot–humid window toward FFA formation and oxidation-susceptible lipid mobilization.

A persistent limitation in postharvest assessment is the reliance on a narrow set of bulk quality indices, even though early treatment responses may reflect coordinated shifts in membrane integrity, lipid hydrolysis, and broader metabolic organization (Pott et al., 2020; Yu et al., 2021). In this context, targeted quantification of free fatty acids (FFAs) provides a direct quantitative readout of early lipid mobilization, whereas untargeted metabolomics offers metabolome-wide context for identifying pathway-associated shifts and treatment-relevant markers. Integrating these two analytical layers therefore makes it possible to evaluate early lipid destabilization not only as an endpoint burden, but also as a process-linked metabolic phenotype. Here, we used a controlled seed-level postharvest model to test whether HPP pretreatment alters early lipid mobilization during a defined hot–humid holding period representing a pre-drying risk window rather than a direct whole-fruit storage simulation. Specifically, freshly harvested *C. oleifera* fruits were first subjected to HPP, after which intact seeds were isolated and exposed to a defined hot–humid holding step. This design allowed us to examine whether HPP shifts the early postharvest metabolic trajectory toward a distinct lipid-mobilization state and to identify pathway-associated markers relevant to front-end process design.

## 2 Materials and methods

### 2.1 Plant materials and postharvest treatments

Fully mature *C. oleifera* fruits were harvested from an experimental plantation in Guangxi, China, shortly after the Shuangjiang (“First Frost”) solar term. Fruits with visible damage were discarded. Intact fruits were vacuum-packaged and subjected to HPP at 100 or 500 MPa for 5 min at the target pressure, or left untreated as controls. HPP was performed using a 15 L ultra-high-pressure food-processing system (HPP600MPa; Baotou Kefa High Pressure Technology Co., Ltd., Baotou, China; maximum working pressure, 600 MPa; rated power, 45 kW), with water used as the pressure-transmitting medium. Real-time sample-core temperature and pressure-transmitting-medium temperature were not recorded by the instrument control system; therefore, no unsupported temperature values are reported. For both pressure treatments, the programmed pressurization time was 240 s. Accordingly, the nominal average pressure-increase rates were approximately 0.42 MPa s⁻¹ for the 100 MPa treatment and 2.08 MPa s⁻¹ for the 500 MPa treatment. The stated 5 min treatment duration refers only to the holding period after the target pressure had been reached (300 s). After the holding step, pressure was released through the instrument-controlled rapid-decompression procedure and returned to ambient pressure almost immediately; no controlled linear depressurization ramp was applied. The pressurization and depressurization steps were therefore not included in the 5 min holding time. Immediately after pressure release, the pericarps were removed and intact seeds, including the seed coat, were collected. Pericarps were removed at this stage to standardize the external conditions experienced during the subsequent holding step, because HPP caused treatment-dependent pericarp injury that could otherwise alter barrier properties, moisture exchange, and oxygen exposure among regimes. The isolated seeds were then held in darkness at 35 °C and 95% relative humidity for 12 h in an artificial climate chamber and subsequently flash-frozen in liquid nitrogen and stored at −80 °C. Four regimes were compared: A, untreated baseline (CK-0 h); B, hot–humid holding only; C, HPP at 100 MPa followed by hot–humid holding; and D, HPP at 500 MPa followed by hot–humid holding. Each regime included three independent biological replicates. Each replicate was prepared as a separate pooled seed batch from approximately 20 fruits, corresponding to approximately 40 intact seeds, collected from multiple independent trees within the same harvest batch. Replicate pools were assembled independently, with no fruit or seed shared among pools.

### 2.2 Targeted profiling of free fatty acids by GC-MS

#### 2.2.1 Sample preparation, extraction, and derivatization

Approximately 50 mg of frozen seed powder was weighed into a 2.0 mL microcentrifuge tube. Samples were extracted with 500 μL of isopropanol:n-hexane (2:3, v/v) containing stearic-d35 acid (0.2 mg L⁻¹) as an internal standard and vortex-mixed for 30 s. The mixture was then homogenized in a bead mill at 40 Hz for 4 min and sonicated for 5 min in an ice-water bath; this homogenization–sonication cycle was repeated three times. After centrifugation at 12,000 rpm for 15 min at 4 °C, approximately 450 μL of the supernatant was transferred to a fresh 1.5 mL tube. The residue was re-extracted with an additional 500 μL of the same extraction solvent containing the internal standard using the same procedure. The supernatants from the two extraction steps were combined and vortexed for 10 s. An 800 μL aliquot of the combined extract was then transferred to a fresh 2.0 mL tube and evaporated to dryness under a gentle stream of nitrogen. For derivatization, 500 μL of methanol:trimethylsilyldiazomethane (1:2, v/v) was added to the dried residue, and the mixture was allowed to react at room temperature for 30 min. After derivatization, the samples were dried again under nitrogen and reconstituted in 160 μL of n-hexane. The solution was centrifuged at 12,000 rpm for 1 min, and the resulting supernatant was transferred to an autosampler vial for GC-MS analysis.

#### 2.2.2 GC-MS instrumentation and operating conditions

GC-MS analysis was performed on an Agilent 7890B gas chromatograph coupled to an Agilent 5977B mass selective detector (Agilent Technologies, Santa Clara, CA, USA). Chromatographic separation was achieved on a DB-FastFAME capillary column (90 m × 250 μm i.d., 0.25 μm film thickness; Agilent J&W, Folsom, CA, USA). A 1 μL aliquot of each derivatized sample was injected in split mode at a split ratio of 5:1. Helium was used as the carrier gas, and the column head pressure was maintained at 46 psi. The oven temperature program was set as follows: initial temperature of 50 °C held for 1 min; increased to 200 °C at 50 °C min⁻¹ and held for 15 min; increased to 210 °C at 2 °C min⁻¹ and held for 1 min; and then increased to 230 °C at 10 °C min⁻¹ and held for 15 min. The injector, transfer line, ion source, and quadrupole temperatures were set at 240, 240, 230, and 150 °C, respectively. Electron ionization was performed at 70 eV. Data were acquired in selected ion monitoring (SIM) mode following a 7 min solvent delay.

#### 2.2.3 Calibration, quantification, and calculation

Quantification of the 49 targeted free fatty acids (FFAs) was carried out using authentic-standard calibration with an FFA standard mixture (mix52; ANPEL, Shanghai, China), with stearic-d35 acid (98%; Sigma-Aldrich, St. Louis, MO, USA) used as the internal standard. Calibration curves for each analyte were constructed by plotting the analyte-to-internal-standard peak-area ratio against the nominal concentration and fitting the data by weighted linear regression using compound-specific weighting factors (1/x or 1/x²). Compound-specific retention times, calibration equations, linear ranges, coefficients of determination (R²), and QC performance metrics are summarized in **Supplementary Table 1**. The concentration of each analyte in the reconstituted solution (C_1_, ng mL⁻¹) was calculated from the corresponding calibration equation, and the FFA content in the sample was calculated as follows:


$$C_{con}=\frac{C_{S}\times V_{1}\times V_{2}}{M\times V_{3}}$$

where *C_con_* is the FFA content on a fresh-weight basis (ng mg^−1^ FW), *Cs* is the analyte concentration in the reconstituted solution (ng mL^−1^), *V_1_* is the reconstitution volume (mL), *V_2_* is the total extraction-solvent volume added (μL), *V_3_* is the extract volume used for derivatization and analysis (μL), and *M* is the sample mass (mg). In this study, *V_1_* = 0.16 mL, *V_2_* = 1000 μL, and *V_3_* = 800 μL. Method performance was monitored by injecting one QC standard sample after every 10 sample injections. QC acceptance criteria were defined as 80%–120% accuracy and a relative standard deviation (RSD) of ≤20%. All 49 targeted FFAs met these acceptance criteria.

### 2.3 Untargeted metabolomics by UHPLC-Orbitrap MS

#### 2.3.1 Metabolite extraction

Plant samples (20 ± 1 mg) were collected, lyophilized, and transferred to tubes containing beads. Each sample was extracted with 1000 μL of extraction solvent [MeOH: ACN:H_2_O, 2:2:1 (v/v/v)] containing deuterated internal standards. The mixture was vortex-mixed for 30 s, homogenized at 35 Hz for 4 min, and sonicated for 5 min in a 4 °C water bath; this homogenization–sonication cycle was repeated three times. The extracts were then incubated at −40 °C for 1 h to precipitate proteins, followed by centrifugation at 12,000 rpm (RCF = 13,800 × g; rotor radius = 8.6 cm) for 15 min at 4 °C. A 400 μL aliquot of the supernatant was then transferred to a well of a protein precipitation plate. The plate was placed on a manifold and processed under vacuum (6 psi) for 120 s, and the resulting filtrate was collected for subsequent analysis. A pooled quality control (QC) sample was prepared by combining equal aliquots of the supernatants from all samples.

#### 2.3.2 LC-MS/MS acquisition (polar and non-polar modes)

For polar metabolites, LC-MS/MS analysis was performed using a Vanquish UHPLC system (Thermo Fisher Scientific) equipped with a Waters ACQUITY UPLC BEH Amide column (2.1 mm × 100 mm, 1.7 μm) and coupled to an Orbitrap Exploris 120 mass spectrometer (Thermo Fisher Scientific). The mobile phase consisted of (A) 25 mmol L⁻¹ ammonium acetate and 25 mmol L⁻¹ ammonium hydroxide in water (pH 9.75) and (B) acetonitrile. The column temperature was maintained at 30 °C, the autosampler was kept at 4 °C, and the injection volume was 2 μL.

For non-polar metabolites, LC-MS/MS analysis was performed on the same UHPLC system fitted with a Phenomenex Kinetex C18 column (2.1 mm × 100 mm, 2.6 μm) and coupled to the same Orbitrap Exploris 120 mass spectrometer. The mobile phase consisted of (A) 0.01% acetic acid in water and (B) IPA: ACN (1:1, v/v). The column temperature was maintained at 25 °C, the autosampler was kept at 4 °C, and the injection volume was 2 μL.

The Orbitrap Exploris 120 mass spectrometer was controlled using Xcalibur (v4.4, Thermo Fisher Scientific) and operated in information-dependent acquisition (IDA) mode, consistent with the vendor-specified workflow. Under this acquisition scheme, full-scan MS spectra were continuously surveyed to trigger MS/MS acquisition. The electrospray ionization (ESI) source parameters were set as follows: sheath gas, 50 Arb; auxiliary gas, 15 Arb; sweep gas, 1 Arb; capillary temperature, 320 °C; vaporizer temperature, 350 °C; full-scan MS resolution, 60,000; MS/MS resolution, 15,000; collision energy, stepped normalized collision energy (NCE) of 20/30/40; and spray voltage, 3.8 kV in positive ion mode and −3.4 kV in negative ion mode.

#### 2.3.3 Data processing, metabolite annotation, and quality control

Raw LC-MS data files were converted to mzXML format using ProteoWizard (v3.0.24054) and processed using an in-house R workflow based on XCMS for peak detection, feature extraction, retention-time alignment, and peak integration. All samples within each chromatographic and ionization-mode dataset were acquired in a single analytical batch; therefore, no inter-batch correction was required. Because of the limited sample number and the single-batch acquisition design, no additional pooled-QC-based intensity drift correction was applied to the exported feature table. No separate solvent-blank- or extraction-blank-subtracted feature table was generated, and blank-based feature filtering was not applied. Instead, analytical consistency was monitored using isotope-labeled internal standards during extraction and repeated injections of an RTQC standard mixture for retention-time stability assessment.

Metabolite annotation was performed through a vendor-assisted workflow using BiotreeDB (v4.0) (Zhou et al., 2022), an in-house authentic-standard library, and the plant-focused BT-Plant library (v1.1). Annotation confidence was categorized according to an MSI-compatible four-level scheme: Level 1, concordant retention time (RT), precursor m/z (MS1), and MS/MS spectra (MS2) relative to an authentic standard; Level 2, putative annotation supported by combined MS1 and MS/MS matching to curated public reference spectra; Level 3, tentative structural or chemical-class assignment supported by MS1/MS2 matching together with predicted RT consistency; and Level 4, unannotated features. Across the present dataset, 179,233 features were detected, of which 1,338, 804, 289, and 176,802 were assigned to MSI Levels 1, 2, 3, and 4, respectively. Chromatographic stability and RT calibration were monitored by repeated injections of an RTQC standard mixture throughout the analytical run. All six RTQC markers showed RT deviations of less than 30 s (1.4–12.0 s), and the post-correction RT model showed excellent agreement between reference and experimental RT values (R² = 0.99; slope = 1.00), indicating stable chromatographic performance and reliable RT-based annotation.

### 2.4 Statistical analysis

Unless otherwise stated, targeted quantitative data are presented as mean ± SEM from three independent biological replicates. The use of three biological replicates per regime is consistent with recent plant and *Camellia*-related metabolomics studies that used three independent biological replicates per treatment, genotype, or sampling point, often in combination with PCA/OPLS-DA and predefined VIP- and fold-change-based screening criteria (Chen et al., 2024; Gong et al., 2025; Wang et al., 2023; Zeng et al., 2023). In the present study, each biological replicate represented an independently pooled seed batch prepared from multiple fruits collected from multiple trees, rather than a single-fruit subsample. Given this sample size, untargeted metabolomics was used as a comparison-level and pathway-contextual layer, whereas targeted FFA profiling was treated as the primary quantitative evidence for lipid mobilization.

Differences among the four treatment regimes for targeted FFA-derived variables, including total FFAs, saturation-class proportions, and dominant-species proportions, were evaluated by one-way ANOVA followed by Tukey’s HSD test. All tests were two-sided, and P < 0.05 was considered significant. For untargeted metabolomics, all statistical analyzes and annotation-derived biological interpretations were performed on the MSI Level 1–2 annotated metabolite matrix; Levels 3 and 4 were retained only for annotation reporting. Hierarchical clustering, PCA, OPLS-DA, pairwise OPLS-DA, differential-metabolite screening, shared-set analysis, recurrence analysis, integrative lipid-context summaries, and the final dual-channel lipid evidence summary were all conducted within this Level 1–2 framework. PCA and OPLS-DA were used to visualize overall treatment-related structure and pairwise metabolic separation. OPLS-DA performance was summarized by R²X(cum), R²Y(cum), and Q²(cum), and model validity was assessed by 200-permutation testing. A metabolite was prioritized as a pairwise discriminant only when all three criteria were satisfied simultaneously: VIP > 1 indicated a meaningful contribution to the OPLS-DA-based separation, |log_2_FC| ≥ 1 required at least a twofold abundance difference between regimes, and P < 0.05 provided nominal univariate statistical support. This combined rule was used to prioritize metabolites with concurrent multivariate importance, effect-size relevance, and statistical evidence, rather than relying on any single criterion alone. Shared discriminants were defined as metabolites meeting these criteria in all three baseline-referenced contrasts (A vs B, A vs C, and A vs D), whereas treatment-specific discriminants were defined as metabolites unique to a single baseline-referenced contrast. Recurrence was quantified as the number of pairwise contrasts in which each metabolite met the same criteria, and recurrent robust markers were defined as metabolites detected in at least five of the six contrasts. For visualization, metabolite abundances were transformed as log2(abundance + 1) and row-wise Z-scored where indicated.

Associations between untargeted signature/module scores and targeted FFA-derived metrics were evaluated using Spearman’s rank correlation, with multiple testing controlled by the Benjamini-Hochberg false discovery rate (BH-FDR) procedure within each analysis family. Chemical-superclass enrichment across discriminant tiers was evaluated by Fisher’s exact test with BH-FDR correction, using the discriminant metabolite space (N = 368) as the background. As a complementary analysis, the targeted FFA abundance matrix was used for multinomial elastic-net classification of the four treatment regimes. Model performance was evaluated by repeated stratified threefold cross-validation with out-of-fold prediction and summarized by Accuracy, Macro-F1, and Macro-AUC. Given the limited sample size, this analysis was interpreted as supportive rather than primary evidence.

## 3 Results

### 3.1 Quality control and confidence-tiered metabolite annotation

**Figure 1:**
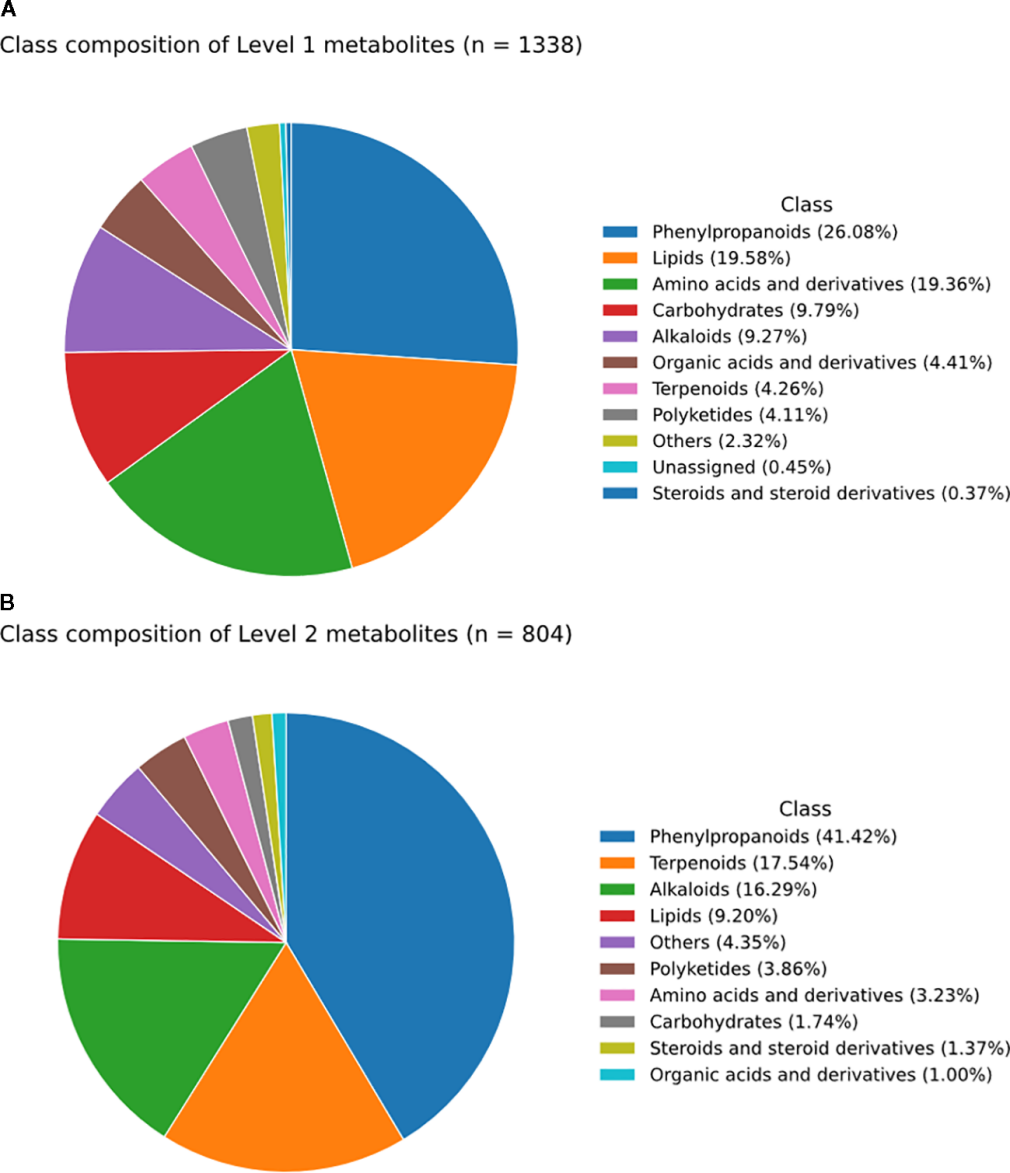
Chemical-class composition of the MSI level 1–2 annotation set in the untargeted LC-MS dataset. **(A)** MSI level 1 metabolites (n = 1,338), confirmed by matching retention time (RT), precursor m/z (MS1), and MS/MS spectra (MS2) to authentic standards. **(B)** MSI level 2 metabolites (n = 804), putatively annotated based on MS1 and MS/MS spectral similarity to curated reference spectra. Metabolites were grouped into major chemical superclasses, and percentages indicate the fraction assigned to each superclass.

Chromatographic performance was monitored using a retention-time quality-control (RTQC) standard mixture. As shown in **Supplementary Figure 1**, the observed retention times closely matched the reference values across repeated RTQC injections (R² = 0.99; slope = 1.00), indicating stable chromatographic performance and supporting reliable retention-time alignment and cross-group comparability in the untargeted LC-MS dataset. **Figure 1** summarizes the chemical-class composition of the high-confidence annotation set. Across MSI Levels 1–2, annotated metabolites spanned ten major superclasses, indicating broad coverage of both primary and specialized metabolism. The Level 1 set (n = 1,338) was enriched in phenylpropanoids (26.08%), lipids (19.58%), and amino acids and derivatives (19.36%), with additional representation from carbohydrates and alkaloids. In contrast, the Level 2 set (n = 804) was dominated by phenylpropanoids (41.42%) and showed greater representation of terpenoids (17.54%) and alkaloids (16.29%). Together, these results show that the high-confidence annotation set captured both lipid-related metabolites and a broad range of stress-responsive phytochemicals relevant to the subsequent analyzes.

### 3.2 Hierarchical clustering reveals treatment-separated metabolic accumulation patterns

**Figure 2:**
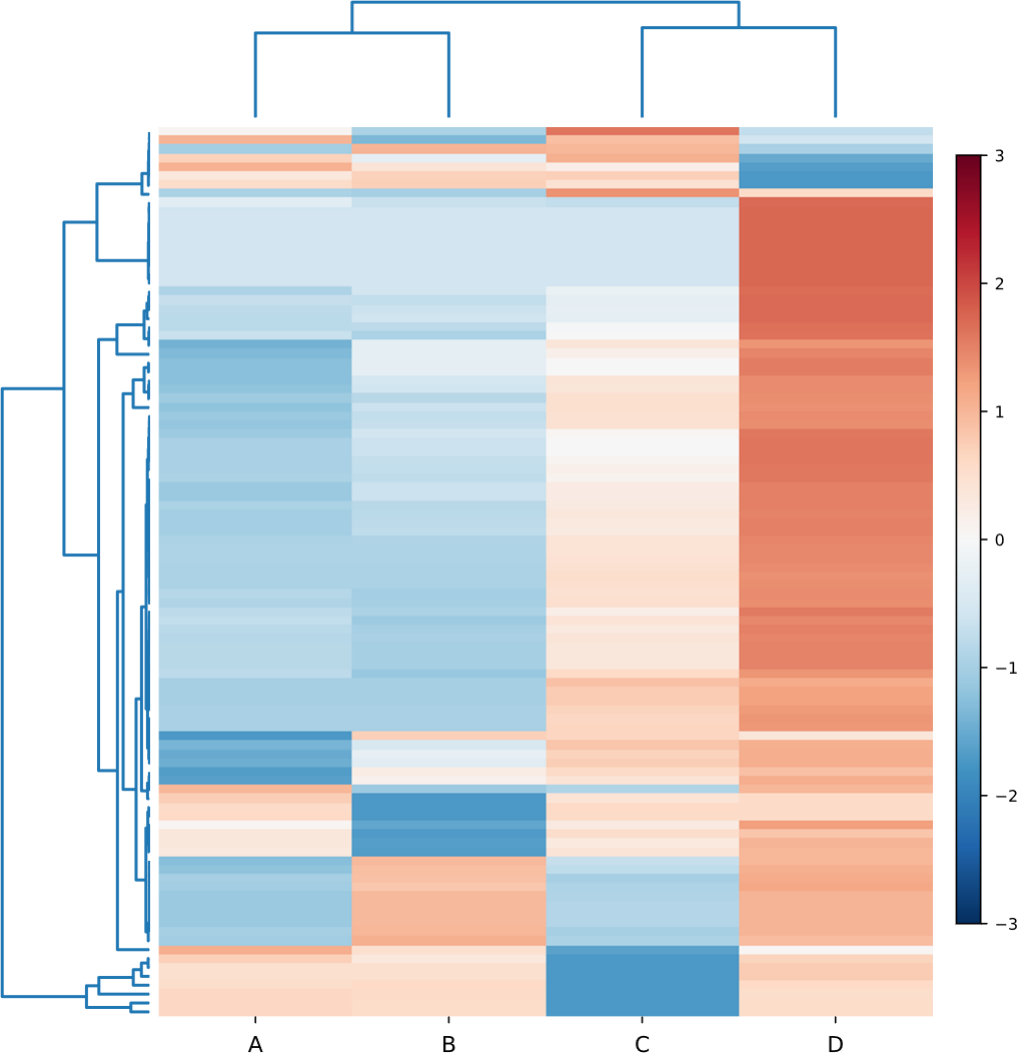
Hierarchical clustering heatmap of the top 100 variable annotated metabolites (MSI levels 1–2) across the four postharvest regimes. Rows represent metabolites and columns represent treatment groups (**(A)**, CK-0 h; **(B)**, hot–humid holding only; **(C)**, HPP at 100 MPa followed by hot–humid holding; **(D)**, HPP at 500 MPa followed by hot–humid holding). Values were row-wise standardized as z-scores. Dendrograms indicate hierarchical clustering of metabolites and treatment groups based on abundance profiles.

To compare global accumulation patterns across treatments, we performed hierarchical clustering of the 100 most variable annotated metabolites (MSI Levels 1–2) and visualized the standardized abundance matrix as a heatmap (**Figure 2**). The sample dendrogram separated the four regimes into two major branches: CK-0 h (A) clustered with hot–humid holding alone (B), whereas the two HPP-pretreated groups (C and D) formed a distinct subcluster. This pattern indicates that pressure pretreatment altered the overall metabolomic configuration beyond the effect of holding alone.

At the metabolite level, the heatmap resolved several coherent modules with contrasting accumulation trajectories. One dominant module showed relatively low abundance in A/B but progressively higher abundance in C and, most prominently, in D, consistent with a pressure-level-associated metabolic shift. In contrast, a smaller module showed reduced abundance specifically in D relative to the other groups, suggesting selective depletion of certain metabolites under the higher-pressure regime. Additional subsets displayed intermediate or group-specific patterns, including changes that were more pronounced in C than in D, indicating that the two pressure treatments did not simply represent scaled versions of the same response. Collectively, these clustering patterns support treatment-associated reconfiguration of metabolite accumulation and provide a focused set of candidates for subsequent comparison-level analyzes.

### 3.3 PCA reveals pressure-level-associated remodeling of the seed metabolome

**Figure 3:**
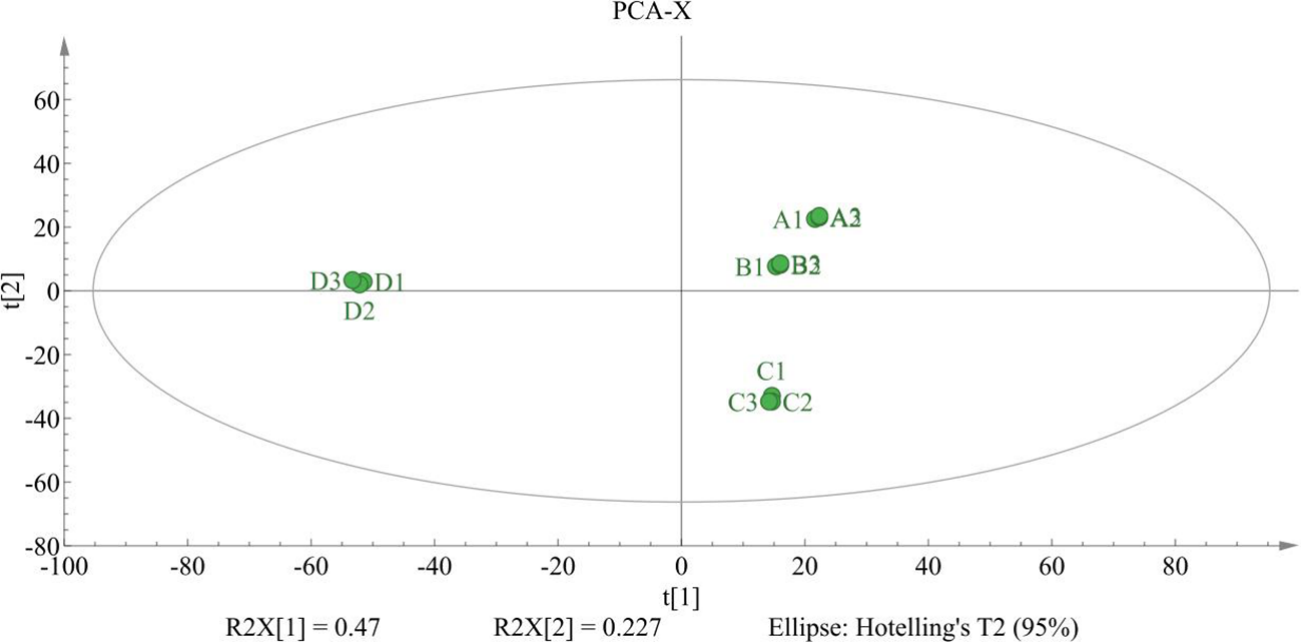
PCA-X score plot of the MSI level 1–2 annotated metabolite matrix across the four postharvest regimes. Each point represents one biological replicate **(A1–A3, B1–B3, C1–C3, and D1–D3)**. The ellipse denotes the 95% Hotelling’s T² confidence region. The first two components explain 47.0% [t(1)] and 22.7% [t(2)] of the variance. **(A)**, CK-0 h; **(B)**, hot–humid holding only; **(C)**, HPP at 100 MPa followed by hot–humid holding; **(D)**, HPP at 500 MPa followed by hot–humid holding.

Principal component analysis (PCA) was applied to the MSI Level 1–2 annotated metabolite matrix to visualize global similarities among samples. The PCA-X score plot revealed clear treatment-dependent structuring of the metabolome (**Figure 3**). Biological replicates clustered tightly within each regime, indicating good within-group reproducibility. Separation along t[1] (R²X[1] = 0.47) was driven primarily by group D, which shifted toward the negative side of the first component relative to groups A–C, indicating that the 500 MPa pretreatment produced the greatest overall multivariate displacement. In contrast, groups A and B occupied closely adjacent positions on the positive side of t[1], suggesting that hot–humid holding alone induced only a modest global shift. Additional separation was observed along t[2] (R²X[2] = 0.227), where group C diverged from the A/B cluster, consistent with an intermediate response at 100 MPa. Together, these PCA patterns support a pressure-level-associated effect on the seed metabolome and provide a multivariate framework for the subsequent identification of discriminant metabolites.

### 3.4 OPLS-DA resolves supervised treatment separation with permutation-based support within the present dataset

**Figure 4:**
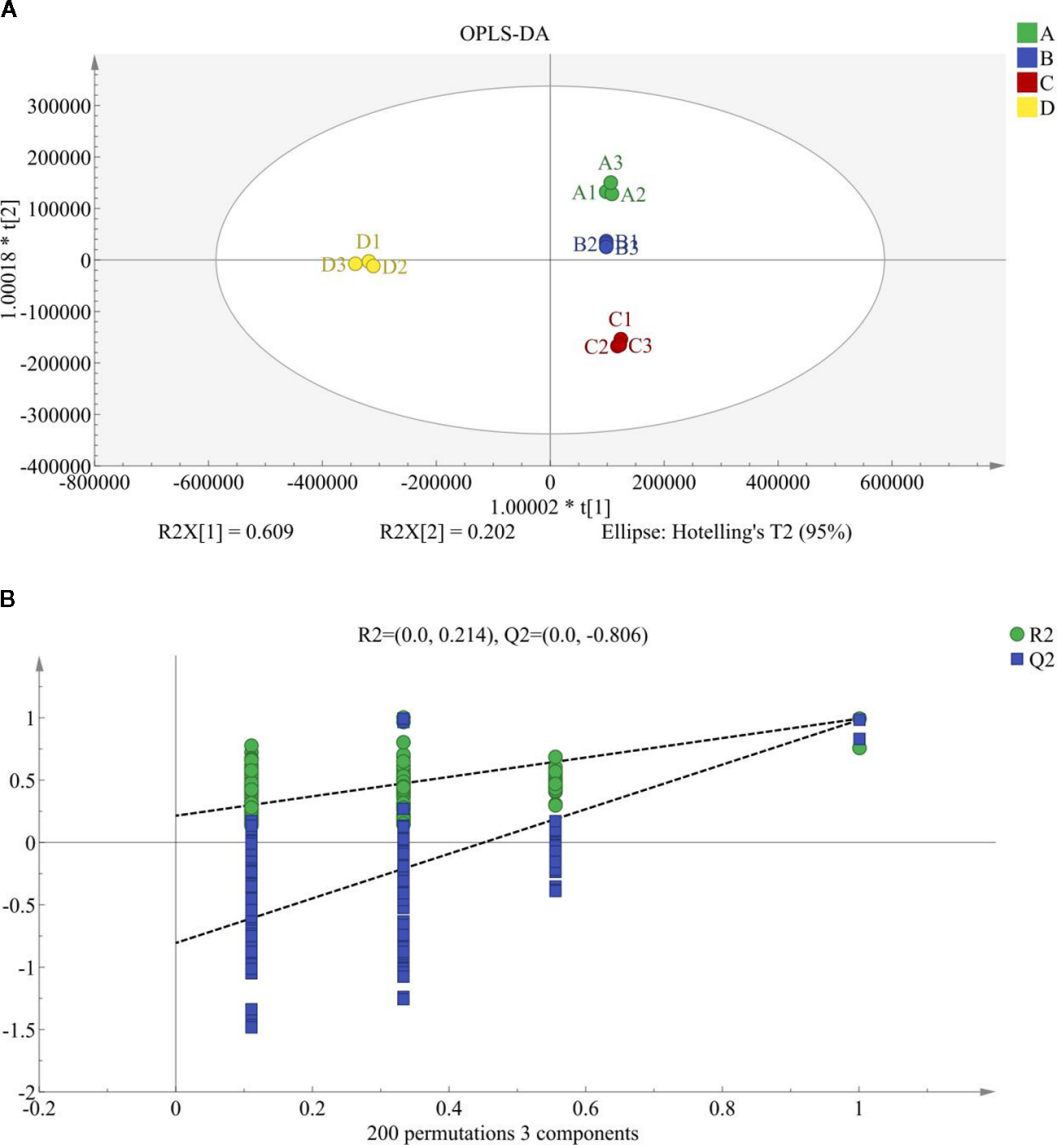
OPLS-DA discrimination of treatment-associated metabolomic profiles and permutation-based model assessment. **(A)** OPLS-DA score plot based on the MSI level 1–2 annotated metabolite matrix, showing separation among regimes **(A–D)**. Each point represents one biological replicate, and the ellipse denotes the 95% Hotelling’s T² confidence region. **(B)** Permutation test of the OPLS-DA model (n = 200). Permuted models yielded markedly lower Q² values than the original model, together with a negative Q² intercept (Q² intercept = −0.806; R² intercept = 0.214), supporting model validity within the present dataset and indicating a low apparent risk of overfitting. Model summary: R^2^X(cum) = 0.917, R^2^Y(cum) = 0.995, and Q^2^(cum) = 0.987.

To further resolve treatment-associated metabolic divergence within a supervised framework and support downstream feature prioritization, an OPLS-DA model was constructed using the MSI Level 1–2 annotated metabolite matrix. The score plot showed clear separation among groups A-D, while biological replicates clustered tightly within each group, indicating reproducible treatment-associated variation within the present dataset (**Figure 4A**). The model showed high explanatory and predictive statistics (R²X(cum) = 0.917; R²Y(cum) = 0.995; Q²(cum) = 0.987). Permutation-based assessment provided additional support for the apparent stability of this supervised separation: the permuted models yielded substantially lower Q² values than the original model and a negative Q² intercept (Q² intercept = −0.806; R² intercept = 0.214), indicating a low apparent risk of overfitting within the present dataset (**Figure 4B**). Accordingly, the OPLS-DA model was used as a supportive, dataset-bound framework for subsequent differential-metabolite screening and comparison-level interpretation under prespecified criteria. Pairwise OPLS-DA score plots and the corresponding 200-permutation tests are shown in **Supplementary Figure 2** (A vs B, A vs C, A vs D, B vs C, B vs D, and C vs D). Together, these untargeted multivariate analyzes indicated that the four regimes were metabolically distinguishable and that the 500 MPa treatment produced the strongest overall perturbation. To determine whether this comparison-level separation was reflected in a direct lipid-relevant phenotype, we next examined targeted free fatty acid (FFA) mobilization as the primary quantitative axis of the study, while using comparison-level discriminant analyzes to contextualize this phenotype.

### 3.5 Pairwise differential metabolite patterns across the four regimes

To place treatment-associated metabolome divergence into a comparison-level framework, we performed six pairwise comparisons (A vs B, A vs C, A vs D, B vs C, B vs D, and C vs D) using unified screening criteria (VIP > 1, |log_2_FC| ≥ 1, and nominal P < 0.05). Thus, the reported discriminant sets represent metabolites that simultaneously contributed to supervised treatment separation, showed a substantial abundance shift, and met the nominal significance threshold in the corresponding pairwise comparison. The resulting discriminant sets varied markedly in size: 113 for A vs B, 138 for A vs C, 216 for A vs D, 128 for B vs C, 195 for B vs D, and 187 for C vs D. Across all six contrasts, the union comprised 368 unique metabolites, indicating that regime-associated metabolic remodeling differed in both breadth and magnitude.

A clear pressure-level-associated pattern emerged. Relative to the untreated baseline, hot–humid holding alone produced the smallest shift, the 100 MPa pretreatment induced an intermediate response, and the 500 MPa pretreatment generated the broadest perturbation. The largest discriminant set was observed for A vs D, indicating that the 500 MPa regime occupied the most distinct metabolic state among the four regimes. A complete comparison-level lookup table is provided in **Supplementary Table 2**.

### 3.6 Shared and treatment-specific discriminants reveal pressure-level-associated signature expansion

**Figure 5:**
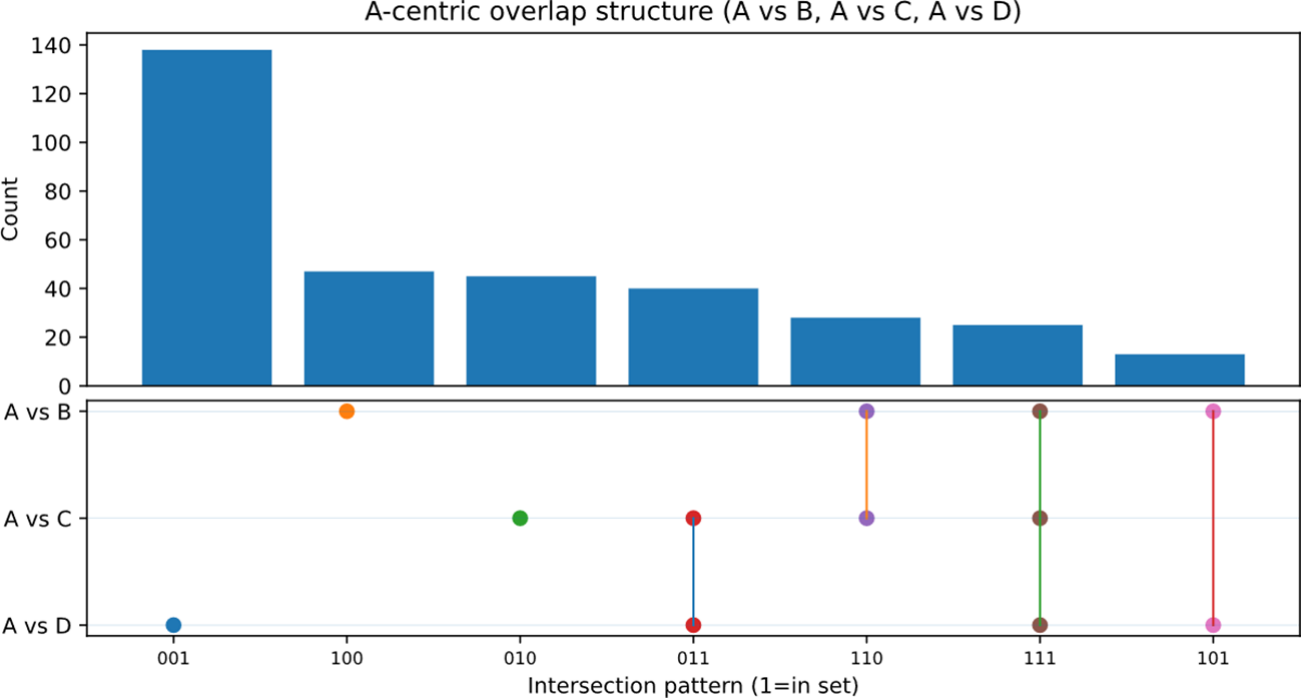
Shared and treatment-specific discriminants across the three baseline-referenced contrasts. UpSet representation of discriminant metabolites identified in A vs B, A vs C, and A vs D using the unified screening criteria of VIP > 1, |log_2_FC| ≥ 1, and nominal P < 0.05. Intersection bars indicate overlap sizes, and the dot matrix indicates set membership across contrasts. The intersection across all three contrasts defines the shared discriminant set Core_A (n = 25), whereas metabolites unique to a single contrast represent treatment-specific discriminants. pairwise and partial overlaps are also shown. The complete metabolite lookup table is provided in **Supplementary Table 2**.

**Figure 6:**
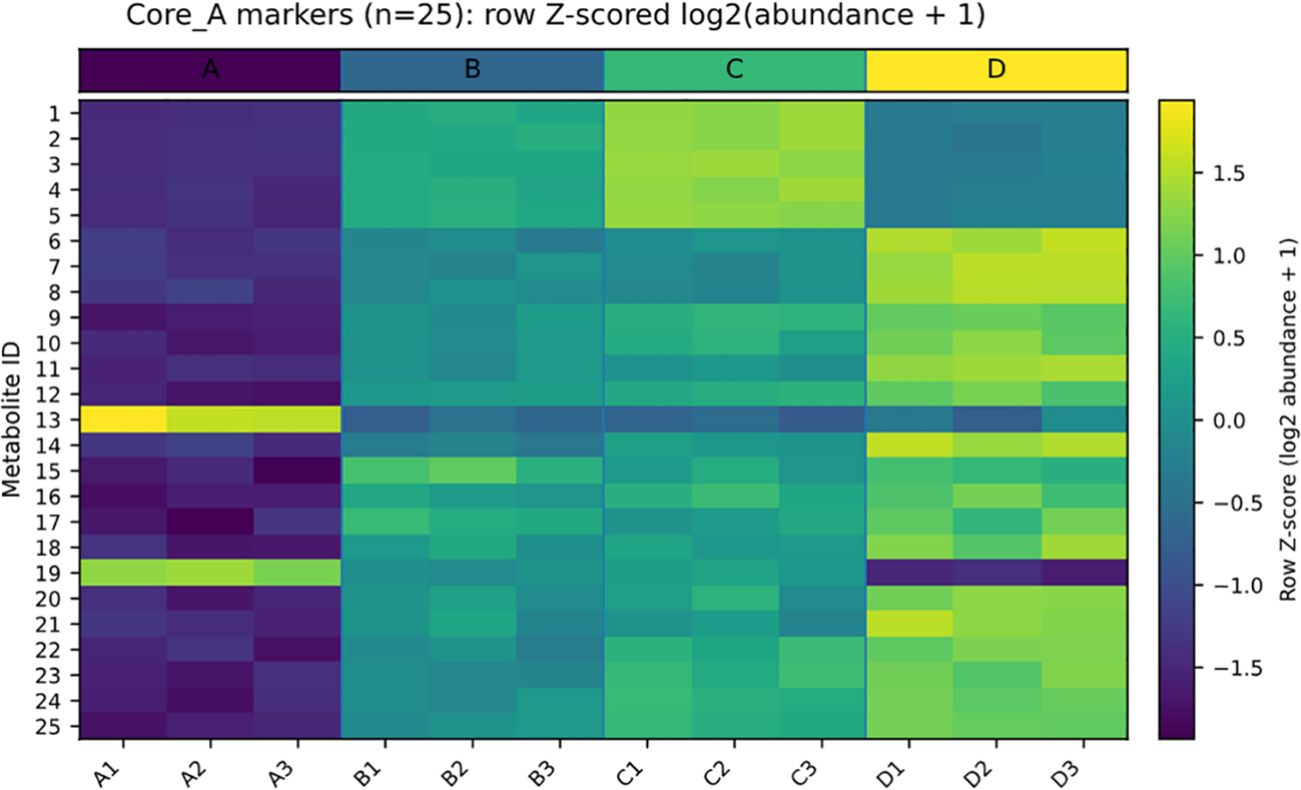
Core_A exhibits coherent and replicate-consistent abundance patterns across regimes. Heatmap of the Core_A metabolites (n = 25) across 12 samples **(A1–A3, B1–B3, C1–C3, and D1–D3)**. Metabolite abundances were transformed as log2(abundance + 1) and row-wise Z-scored to emphasize relative abundance patterns across regimes. Metabolites are displayed as numeric IDs, and the corresponding ID-to-name mapping is provided in the accompanying legend table. The top annotation strip indicates regime identity.

We next reorganized the discriminant space across the three baseline-referenced contrasts (A vs B, A vs C, and A vs D) to distinguish shared from treatment-specific signals under the same hot–humid window (**Figure 5**; **Table 1**; **Supplementary Table 3**). This analysis identified a shared core discriminant set (Core_A; n = 25), together with B-, C-, and D-specific sets comprising 47, 45, and 138 metabolites, respectively. To improve readability, the main-text **Table 1** reports only the key annotation, chemical-class, direction-consistency, and compact statistical summaries for the Core_A metabolites, whereas detailed log_2_FC ranges and contrast-level statistics are provided in **Supplementary Table 3A**. When mapped back onto the abundance matrix, the Core_A metabolites displayed coherent and replicate-consistent patterns across regimes (**Figure 6**), supporting their interpretation as a stable shared response layer. In parallel, the substantially larger D-specific set indicated that the 500 MPa regime was distinguished not only by greater overall divergence, but also by stronger expansion of treatment-specific signatures. Thus, under a common hot–humid holding condition, pressure primarily enlarged the unique component of the metabolome response rather than simply amplifying a shared background shift.

**Table 1:** Core_A shared discriminant metabolites identified across the three A-referenced contrasts.

Metabolite ID	Metabolite	Super.Class	Class	Direction consistency	max_VIP (A vs B/C/D)	min_p (A vs B/C/D)
1	4-Ethoxybenzoic acid	Phenylpropanoids	Phenolic acids	Consistent	5.288	6.77 × 10⁻^6^
2	Ethyl 4-hydroxybenzoate	Phenylpropanoids	Phenolic acids	Consistent	5.262	3.18 × 10⁻^5^
3	Methyl 4-hydroxy-2-methylbenzoate	Phenylpropanoids	Phenolic acids	Consistent	5.248	1.72 × 10⁻^5^
4	2-Phenyllactic acid	Phenylpropanoids	Other Phenylpropanoids	Consistent	5.244	6.85 × 10⁻^6^
5	Ethyl 3-hydroxybenzoate	Phenylpropanoids	Phenolic acids	Consistent	5.109	1.30 × 10⁻^5^
6	Isoleucine	Amino acids and derivatives	Aminoacids	Consistent	4.324	3.26 × 10⁻^5^
7	D-Leucine	Amino acids and derivatives	Amino acid derivatives	Consistent	4.316	4.37 × 10⁻^5^
8	Leucine	Amino acids and derivatives	Aminoacids	Consistent	4.207	9.50 × 10⁻^6^
9	Erucamide	Lipids	Fatty Acids and Conjugates	Consistent	3.926	8.81 × 10⁻^6^
10	Pyridoxine	Alkaloids	Pyridine alkaloids	Consistent	3.48	1.08 × 10⁻^4^
11	Proline	Amino acids and derivatives	Aminoacids	Consistent	3.331	1.49 × 10⁻^5^
12	(E)-3,4-Dimethoxycinnamic acid	Phenylpropanoids	Other Phenylpropanoids	Consistent	3.126	8.07 × 10⁻^6^
13	(1S,4aR,6aS,6bR,10R,11R,12aR)-1,10,11-trihydroxy-9,9-bis(hydroxymethyl)-2,2,6a,6b,12a-pentamethyl-1,3,4,5,6,6a,7,8,8a,10,11,12,13,14b-tetradecahydropicene-4a-carboxylic acid	Terpenoids	Triterpenoids	Consistent	2.542	2.81 × 10⁻^4^
14	Methionine	Amino acids and derivatives	Aminoacids	Consistent	2.092	5.00 × 10⁻^5^
15	Phlorizin	Phenylpropanoids	Flavonoids	Consistent	1.928	1.22 × 10⁻^4^
16	2-Propenoic acid, 3-(3,4-dimethoxyphenyl)-	Phenylpropanoids	Other Phenylpropanoids	Consistent	1.912	1.79 × 10⁻^4^
17	beta-Alanine	Amino acids and derivatives	Aminoacids	Consistent	1.743	1.83 × 10⁻^4^
18	o-Tyrosine	Amino acids and derivatives	Amino acid derivatives	Consistent	1.627	1.37 × 10⁻^4^
19	5-Hydroxy-6’’,6’’-dimethylpyrano[2’’,3’’:7,8]flavone	Phenylpropanoids	Flavonoids	Consistent	1.598	1.86 × 10⁻^4^
20	Tyrosine	Amino acids and derivatives	Aminoacids	Consistent	1.591	1.33 × 10⁻^5^
21	proline dl-form	Amino acids and derivatives	Amino acid derivatives	Consistent	1.483	1.28 × 10⁻^4^
22	N-Methylanthranilic acid	Alkaloids	Other alkaloids	Consistent	1.475	4.98 × 10⁻^5^
23	Methyl 5-methylpyridine-3-carboxylate	Alkaloids	Other alkaloids	Consistent	1.469	7.80 × 10⁻^5^
24	(2-Nitroethyl)benzene	Phenylpropanoids	Other Phenylpropanoids	Consistent	1.43	1.76 × 10⁻^5^
25	N-Phenylglycine	Amino acids and derivatives	Amino acid derivatives	Consistent	1.404	6.18 × 10⁻^6^

Core_A comprises metabolites that met the unified screening criteria of VIP > 1, |log_2_FC| ≥ 1, and P < 0.05 in all three A-referenced contrasts: A vs B, A vs C, and A vs D. The table reports metabolite annotations, chemical classes, direction consistency across the three contrasts, and compact statistical summaries. Direction consistency indicates whether each metabolite showed a concordant direction of change relative to A across the three comparisons. Max_VIP and Min_P denote the maximum VIP value and the minimum nominal P value observed across A vs B, A vs C, and A vs D, respectively. Detailed log_2_FC ranges and contrast-level statistics for the Core_A metabolites are provided in **Supplementary Table 3A**.

### 3.7 Recurrent robust markers across comparisons

**Figure 7:**
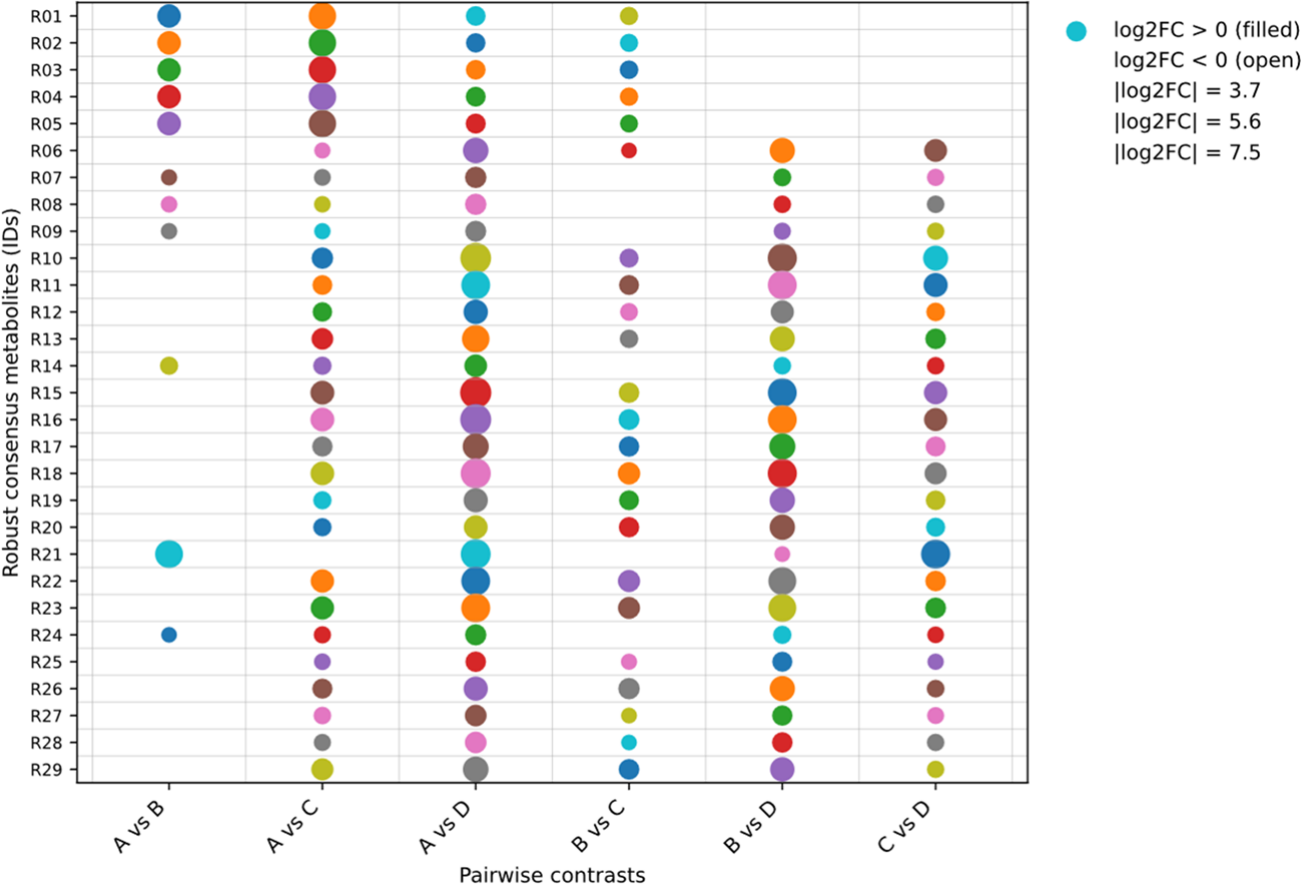
Recurrent robust markers across the six pairwise contrasts. Dot plot of the recurrent robust markers detected in at least five of the six pairwise comparisons. Each row represents one metabolite (R01–R29), and columns denote the six contrasts (A vs B, A vs C, A vs D, B vs C, B vs D, and C vs D). Dot size scales with absolute effect size (|log_2_FC|), and dot fill indicates directionality (filled, log_2_FC > 0; open, log_2_FC < 0). Missing dots indicate the single contrast in which a metabolite did not meet the unified screening criterion (VIP > 1, |log_2_FC| ≥ 1, and nominal P < 0.05) for inclusion in the 5/6 recurrence tier. A complete lookup table, including chemical annotation, the identity of the missing contrast (if any), and summary statistics, is provided in **Supplementary Table 4**.

We next quantified recurrence across all six pairwise contrasts to identify metabolites that were reproducibly selected beyond the baseline-referenced framework. Recurrence was highly uneven across the 368-metabolite union: 87 metabolites appeared in only one contrast, whereas 29 were detected in at least five of the six contrasts, including five present in all six (**Figure 7**; **Supplementary Table 4**). This recurrent marker set captures a complementary dimension of the comparison-wide response. Whereas the shared discriminants described in Section 3.6 represent metabolites consistently selected across the three baseline-referenced contrasts, the recurrent robust markers identify metabolites that remained reproducibly detectable across the broader six-contrast framework. Together, these results indicate that the untargeted response comprised both a stable shared component and a more selective layer of broadly recurrent markers. Rather than representing independent descriptive endpoints, these shared, treatment-specific, and recurrent metabolite tiers define the regime-structured metabolic background against which the targeted FFA phenotype was evaluated. The expansion of D-specific and broadly recurrent signatures, in particular, provided a comparison-level rationale for testing whether the strongest metabolome divergence converged on a quantitative lipid-mobilization axis.

### 3.8 Targeted FFA profiling defines the primary quantitative lipid axis

**Figure 8:**
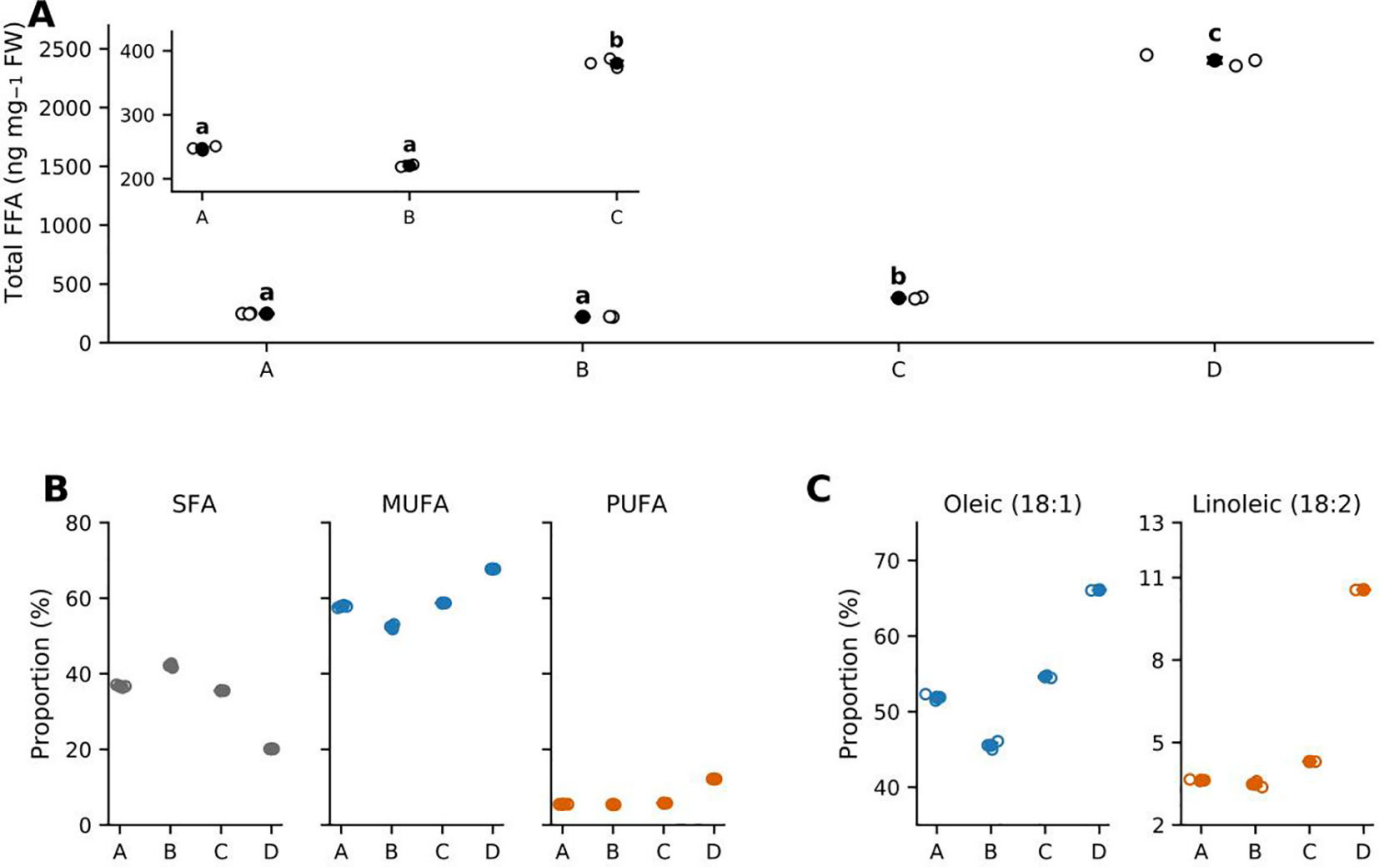
Targeted free fatty acid profiling reveals regime-dependent shifts in total FFA burden and unsaturation. **(A)** Total free fatty acid (FFA) abundance across regimes **(A–D)**, expressed on a fresh-weight basis (ng mg⁻¹ FW). Open circles denote individual biological replicates (n = 3), and filled circles indicate mean ± SEM. Different letters indicate significant differences according to Tukey’s HSD following one-way ANOVA (P = 8.56 × 10⁻¹^4^). The inset provides a magnified view of regimes A–C. **(B)** Saturation-class composition of the detected FFA pool, shown as the proportions (%) of saturated FFAs (SFA), monounsaturated FFAs (MUFA), and polyunsaturated FFAs (PUFA). Open circles denote individual biological replicates (n = 3), and filled circles indicate mean ± SEM. The combined unsaturated fraction (MUFA + PUFA) differed significantly among regimes (one-way ANOVA, P = 3.27 × 10⁻¹²). **(C)** Proportions (%) of the dominant species oleic acid (C18:1) and linoleic acid (C18:2) across regimes. Open circles denote individual biological replicates (n = 3), and filled circles indicate mean ± SEM. See **Supplementary Table 1** for the target list, calibration metrics, and replicate-level quantification.

To establish the primary quantitative evidence for treatment-associated lipid mobilization, we quantified 49 targeted free fatty acids (FFAs) across the four regimes (A–D; n = 3 biological replicates per regime). Of these, 42 FFAs were detected in at least one regime, whereas seven were undetectable across all samples (**Supplementary Table 1**). Within-regime reproducibility was excellent, with median coefficients of variation of approximately 2% and all values below 3% for the detected species, supporting reliable between-regime comparisons within the targeted FFA dataset. Total FFA abundance differed markedly among regimes (one-way ANOVA, P = 8.56 × 10⁻¹^4^; **Figure 8A**). Regimes A and B remained broadly comparable (A: 247.50 ± 1.95 ng mg⁻¹; B: 220.47 ± 1.01 ng mg⁻¹, mean ± SEM), indicating that hot–humid holding alone did not substantially increase the total free-acyl burden within the present time window. In contrast, regime C showed a moderate increase (380.59 ± 4.17 ng mg⁻¹), whereas regime D exhibited a pronounced step-change elevation (2401.41 ± 26.61 ng mg⁻¹), clearly separating the 500 MPa treatment from the other three regimes. Thus, under the present postharvest model, the primary quantitative effect of HPP was a pressure-level-dependent lipid-mobilization response, with the strongest phenotype observed at 500 MPa.

The composition of the FFA pool also shifted across regimes (**Figures 8B, C**). The combined unsaturated fraction (MUFA + PUFA) increased from 63.29% in A to 79.87% in D, indicating that the elevated free-acyl burden in the 500 MPa regime was enriched in unsaturated species rather than reflecting a proportional increase across all FFAs. Oleic acid (18:1) remained the dominant free fatty acid in all regimes, and its proportional contribution increased further under pressure pretreatment, particularly in D. Linoleic acid (18:2) also increased, whereas the relative contribution of saturated FFAs declined. Together, these compositional data indicate that the pressure-associated increase in total FFAs was accompanied by a shift toward an unsaturation-enriched free-acyl pool. At the species level, the strongest treatment-associated increases were concentrated in a subset of major C18 and related unsaturated FFAs, whereas many minor species showed smaller or less consistent responses (**Supplementary Table 1**). This pattern indicates that the treatment effect was structured rather than diffuse, with the 500 MPa regime amplifying a specific lipid-mobilization phenotype rather than uniformly elevating the entire detectable FFA background. Collectively, these targeted results establish the primary quantitative lipid axis of the study, with the 500 MPa regime representing the strongest and most distinct lipid-mobilization phenotype. This FFA increase is reported here as an early lipid-mobilization signal and should not be interpreted, at the Results stage, as evidence of improved oil quality. We therefore used this targeted FFA axis as the central quantitative reference for the integrative untargeted analyzes presented below.

### 3.9 Selective convergence of untargeted metabolome signatures on the targeted FFA phenotype

**Figure 9:**
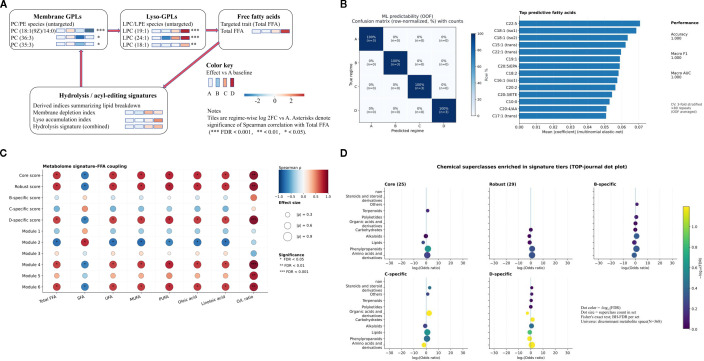
Alignment of untargeted metabolome-derived signatures with the targeted free-fatty-acid (FFA) axis. **(A)** Integrated, regime-aligned summary of lipid-mobilization-related readouts. Representative membrane glycerophospholipids (GPLs) and lyso-glycerophospholipids (Lyso-GPLs) are shown as tiles [log2 fold-change relative to regime A], together with targeted total FFA abundance and composite indices summarizing hydrolysis- versus acyl-editing-related tendencies. Asterisks denote significant Spearman correlations with total FFA after BH-FDR correction. **(B)** Out-of-fold classification of the four regimes using the targeted FFA abundance matrix. Left, row-normalized confusion matrix (%) with counts; middle, top predictive fatty acids ranked by mean absolute coefficient magnitude from a multinomial elastic-net model; right, cross-validated performance metrics (Accuracy, Macro-F1, and Macro-AUC) obtained by repeated stratified threefold cross-validation. Given the limited sample size, this analysis is presented as supportive rather than primary evidence. **(C)** Spearman correlation dot-heatmap linking metabolome-derived signature scores and module scores to FFA-derived metrics (total FFA; SFA, MUFA, and PUFA fractions; oleic acid; linoleic acid; and O/L ratio). Dot color indicates Spearman’s ρ, and dot size indicates |ρ|. Statistical significance was evaluated using BH-FDR correction, with significance thresholds indicated in the key. Source data: **Supplementary Table 5**. **(D)** Chemical-superclass enrichment across shared, recurrent, and treatment-specific discriminant sets (numbers in parentheses indicate metabolite counts per set). The x-axis shows log2(odds ratio), dot size represents superclass counts in each set, and dot color encodes −log10(BH-FDR). Enrichment was assessed by Fisher’s exact test with BH-FDR correction within each set, using the discriminant-metabolite space defined in this study (N = 368) as the background universe. “nan” denotes features lacking superclass annotation.

Having established targeted FFA profiling as the primary quantitative lipid axis of this study, we next examined how untargeted metabolome-wide signatures aligned with that phenotype across the four regimes (A–D). To address this question, we integrated the shared, recurrent, and treatment-specific discriminant tiers with targeted FFA-derived readouts in a regime-oriented summary (**Figure 9A**). This summary combines representative membrane glycerophospholipid (GPL) and Lyso-GPL features, total FFA burden and selected FFA-derived metrics, and composite indices intended to reflect hydrolysis- versus acyl-editing-related tendencies. Across regimes, these lipid-associated readouts varied in both magnitude and direction, indicating that the pressure-level-associated FFA phenotype was embedded within a broader yet structured metabolome response rather than representing an isolated targeted endpoint. To provide pathwaS3y-level context for the unsaturation shift identified by targeted FFA profiling, we further mapped the major unsaturated fatty acids (18:1 → 18:2 → 18:3), together with representative oxylipin- and jasmonate-related mediators, onto a simplified remodeling-and-oxygenation scheme (Figure). Relative to the A baseline, the HPP-primed hot–humid holding regimes, particularly D, showed coordinated increases in 18:2, 18:3, and selected downstream mediators, whereas B showed comparatively weaker or downward shifts at several nodes. These patterns were descriptive rather than flux-resolved and therefore provide pathway-level context for the unsaturation-enriched FFA phenotype observed under the 500 MPa regime. Accordingly, we interpreted the oxylipin- and jasmonate-related features as oxygenation-associated metabolic signals rather than as direct evidence of oxidative cleavage or as a basis for quantitatively partitioning the FFA increase into hydrolytic and oxidative components.

We next examined whether regime identity could be recovered from the targeted FFA matrix alone within the present dataset (**Figure 9B**). Under repeated stratified threefold cross-validation with out-of-fold evaluation, classification performance remained high, and multinomial elastic-net coefficients highlighted a subset of fatty acids with stronger discriminatory weight. Given the limited sample size, this analysis was interpreted as supportive rather than primary evidence; nevertheless, it indicates that the targeted FFA phenotype was sufficiently structured to capture regime-level differences within the current experimental design.

At the sample level, we then tested whether untargeted signature organization was statistically coupled to the targeted FFA phenotype space (**Figure 9C**; source data in **Supplementary Table 5**). Spearman correlation analysis showed that selected signature and module scores were associated with total FFA burden and saturation/unsaturation-related metrics, including the O/L ratio, with significance retained after BH-FDR correction. In contrast, other signature-metric pairs remained weak, nonsignificant, or directionally discordant, indicating that convergence between untargeted signatures and targeted lipid metrics was selective rather than uniform across all tiers and modules. Finally, we examined whether the discriminant tiers differed in superclass-level chemical composition (**Figure 9D**). Fisher’s exact tests with BH-FDR correction revealed tier-dependent superclass enrichment patterns, indicating that regime-associated variation was detectable not only along the targeted FFA axis, but also in the chemical-class structure of the discriminant metabolite space. Collectively, these analyzes position the targeted FFA phenotype as the quantitative center of the study while showing that untargeted metabolomics aligned with that phenotype in a selective, regime-structured manner.

### 3.10 Dual-channel lipid evidence for candidate membrane-lipid remodeling

**Figure 10:**
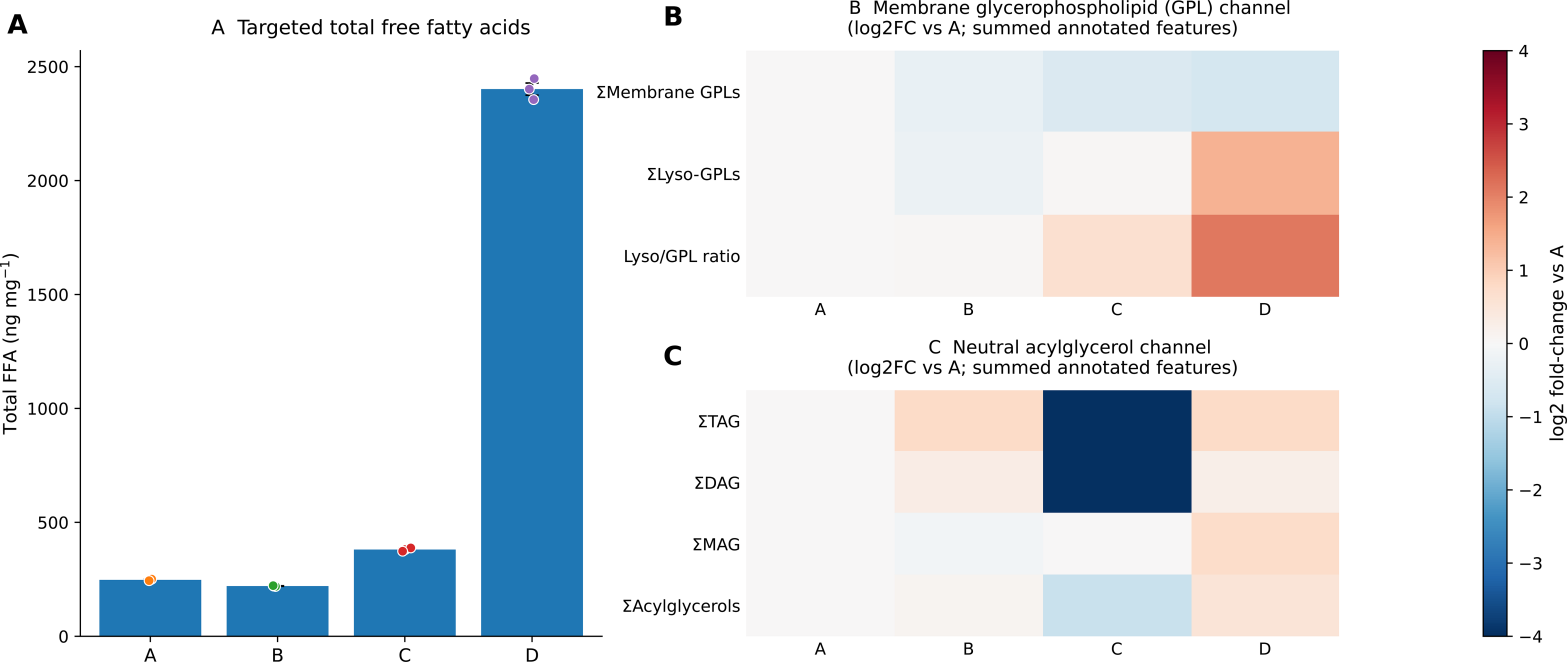
Dual-channel lipid summaries aligned with targeted total FFA elevation. **(A)** Targeted total free fatty acids (FFAs) across regimes **(A–D)** (n = 3 biological replicates per regime); bars show mean ± SEM with individual points overlaid. **(B)** Membrane glycerophospholipid (GPL) channel summarized from the annotated lipidome: regime-wise sums of membrane GPLs (PC/PE/PG/PI/PS/PA/CL) and Lyso-GPLs, and the Lyso/GPL ratio, displayed as log_2_ fold-changes relative to the A baseline. **(C)** Neutral acylglycerol channel summarized from the annotated lipidome: regime-wise sums of annotated TAG, DAG, MAG and their combined acylglycerol total, displayed as log_2_ fold-changes relative to **(A)** Heatmaps are clipped at ±4 for display. Channel summaries in **(B, C)** are computed from the annotated lipid subset, and annotation coverage for each channel is reported in the source data. Source data: **Supplementary Table 6**. The GPL and Lyso-GPL summaries are based on annotation-derived, semi-quantitative untargeted lipid features and are intended to provide lipid-contextual support rather than absolute lipid quantification or causal evidence for a specific FFA-generating pathway.

Having established a pressure-level-associated targeted FFA phenotype and its selective alignment with untargeted metabolome signatures (Sections 3.8–3.9), we next asked whether the elevated FFAs, particularly under the 500 MPa regime, were more consistent with membrane-lipid remodeling or with signals arising from annotated neutral acylglycerol pools. Because these lipid-class summaries were derived from relative intensities of confidence-ranked untargeted annotations, they should be interpreted as regime-aligned, semi-quantitative evidence rather than as absolute lipid quantification or direct evidence of pathway flux. We therefore adopted a deliberately conservative dual-channel summary to evaluate plausible lipid sources underlying the FFA increase (**Figure 10**). In the membrane channel, annotated membrane glycerophospholipids (GPLs; PC, PE, PG, PI, PS, PA, and CL), Lyso-GPLs, and the Lyso/GPL ratio were summarized as regime-wise totals and expressed as log2 fold-changes relative to regime A (**Figure 10B**). Across regimes, membrane GPL-associated signals decreased, whereas Lyso-GPL-associated signals and the Lyso/GPL ratio increased, with the most pronounced shift observed in D. These coordinated directional changes are consistent with enhanced membrane-lipid deacylation and/or remodeling under the same regime in which total FFAs were highest. Importantly, this interpretation is based on directional concordance rather than mass-balance closure, because the summary is restricted to the annotated lipid subset represented in the present dataset.

In parallel, we summarized the annotated neutral acylglycerol channel, including TAG, DAG, MAG, and their combined total, using the same regime-aligned framework (**Figure 10C**). Signals in this channel also varied across regimes, providing a complementary view of possible acyl mobilization. However, because annotation coverage differed across lipid classes and remained more limited for some neutral-acylglycerol components than for the membrane GPL channel, the neutral channel was interpreted as supportive rather than definitive evidence for neutral-lipid involvement. For transparency, **Supplementary Figure 4** provides an audit view of the dual-channel summary, including raw regime-wise membrane-channel totals shown on a log10 scale together with the corresponding log2 fold-change displays and the targeted total FFA readout (source data in **Supplementary Table 6**). Taken together, the dual-channel evidence does not establish a closed mechanistic route, but it does support a conservative, regime-aligned interpretation in which the strongest FFA elevation under 500 MPa is more consistent with membrane GPL remodeling/deacylation than with a dominant signal arising solely from the currently annotated neutral acylglycerol pool. Thus, this analysis supports membrane-lipid remodeling as a plausible contributor to elevated FFAs while explicitly acknowledging the annotation-coverage limits of the present dataset.

Together, the dual-channel summary placed the targeted FFA increase under the 500 MPa regime in a broader lipid-class context. This regime was characterized by lower annotation-derived membrane GPL signals and higher Lyso-GPL-related signals, a directionally coherent pattern with membrane-lipid remodeling as one candidate contributor to the expanded free-acyl pool. Because the GPL and Lyso-GPL channels were calculated from annotation-derived, semi-quantitative untargeted features rather than from absolute lipid measurements, they are best viewed as lipid-contextual support for this candidate pathway. These data therefore do not resolve the relative contribution of membrane remodeling versus neutral acylglycerol hydrolysis or other acyl-release routes to FFA accumulation.

## 4 Discussion

In this study, targeted FFA profiling provided the primary quantitative evidence that HPP converted a relatively modest hot–humid postharvest response into a pressure-level-dependent lipid-mobilization phenotype, whereas untargeted metabolomics supplied broader comparison-level context and conservative mechanistic support. This evidence hierarchy is important because it anchors the main conclusion in a direct, auditable lipid-scale readout, while using metabolome-wide information to interpret, rather than overstate, the underlying biochemical organization.

### 4.1 Pressure priming shifts a short hot–humid window into a pressure-level-dependent lipid-mobilization regime

Targeted FFA profiling showed that HPP pretreatment shifted a short hot–humid holding window into a pressure-level-dependent lipid-mobilization regime in oil-tea seeds (**Figure 8**; **Supplementary Table 1**). Under hot–humid holding alone, the global FFA burden changed only modestly, whereas HPP converted the same window into a clear pressure-associated response, with a moderate increase at 100 MPa and a pronounced increase at 500 MPa. In parallel, the mobilized pool shifted toward higher unsaturation, indicating that pressure altered both the extent and composition of the free-acyl response. This compositional shift indicates that the 500 MPa response should be viewed as a net lipid-mobilization phenotype rather than as a single-process readout. The targeted GC-MS data directly established an expansion of the free-acyl pool, but they did not identify the specific upstream reactions responsible for that expansion. Increased FFAs are consistent with hydrolytic acyl release from complex lipids, particularly under conditions in which pressure may increase membrane permeability and enzyme–substrate accessibility. At the same time, the enrichment of unsaturated C18 FFAs, together with the descriptive changes in selected oxylipin- and jasmonate-related features, suggests that oxygenation-associated metabolism of unsaturated substrates may have occurred in parallel. Thus, the 500 MPa phenotype is best interpreted as the combined outcome of pressure-associated acyl release and possible downstream oxidation-related remodeling, rather than as evidence for pure hydrolysis alone. Because primary lipid oxidation products, including lipid hydroperoxides, conjugated dienes, peroxide value, or malondialdehyde, were not directly quantified, the present dataset cannot determine the quantitative contribution of oxidative cleavage to the observed FFA pool. Importantly, this increase should not be interpreted as an inherently favorable HPP response. Because acid value, peroxide value, primary oxidation products, and extracted-oil sensory quality were not directly measured, the elevated FFA burden is best regarded as an early quality-relevant lipid-destabilization signal rather than direct evidence that the tested HPP regimes either improved or compromised commercial oil quality. Because the pericarps were removed after HPP and before the hot–humid holding step, the present system is best interpreted as a controlled seed-level postharvest model rather than a direct simulation of whole-fruit commercial storage.

This gated behavior is consistent with previous Camellia postharvest studies showing that moisture, temperature, and duration jointly influence deterioration-related outcomes (Zhu et al., 2020, 2024), and that handling choices during drying and storage can either suppress or accelerate lipid-hydrolysis- and oxidation-related changes (Kong et al., 2025; Zhang et al., 2019). Our results add a sequence-sensitive dimension to this literature: a brief physical pretreatment markedly amplified lipid mobilization within an otherwise modest 12 h hot–humid window, with a sharp separation between 100 and 500 MPa. In contrast to pretreatments that may simultaneously disrupt microstructure, reduce lipase/LOX activities, and improve oxidative stability (Chen et al., 2024; Luo et al., 2025; Wu et al., 2025), HPP is typically applied under ambient-to-mild thermal conditions. We therefore infer that HPP may have perturbed cellular or subcellular compartmentation without fully suppressing enzymatic capacity, thereby increasing opportunities for enzyme–substrate contact during the subsequent warm, high-humidity holding period. This interpretation is consistent with general pressure effects on noncovalent interactions, membrane integrity, and post-pressurization cellular damage (Medina-Meza et al., 2014). Although enzyme activities were not measured in the present study, the regime pattern supports the concept of a pressure-primed postharvest sensitivity window.

### 4.2 Pressure priming may couple acyl release with membrane-lipid remodeling during hot–humid holding

The step-change increase in total FFA burden in regime D, together with the enrichment of unsaturated C18 species in the mobilized pool (**Figure 8**), is consistent with a pressure-level-dependent shift in the balance between acyl release and lipid-remodeling capacity during the short hot–humid window. In the regime-aligned integration (**Figure 9A**), targeted FFA metrics shifted in parallel with representative membrane GPL and Lyso-GPL features, as well as with composite indices summarizing hydrolysis- versus acyl-editing-related tendencies, a pattern consistent with acyl release exceeding reacylation and structural buffering after 500 MPa pretreatment. The unsaturation-weighted character of the response provides further context: in oilseeds, unsaturated C18 acyl chains are extensively handled through phosphatidylcholine-centered acyl-editing and exchange processes, and an unsaturation-enriched free-acyl pool is compatible with perturbation of membrane-associated remodeling routes (Bates et al., 2012).

Accordingly, the dual-channel lipid summary should frame, rather than finalize, the mechanistic interpretation of the FFA phenotype. The targeted GC-MS results quantified the expansion of the free-acyl pool, whereas the GPL and Lyso-GPL trends were derived from annotation-based, semi-quantitative untargeted lipid features. A cautious interpretation is that pressure exposure may have relaxed compartmental constraints in the seed matrix, thereby increasing the likelihood that pre-existing endogenous lipid-hydrolyzing enzymes encountered membrane lipid substrates during the subsequent hot–humid holding step. Within this scenario, phospholipase-mediated GPL hydrolysis would be compatible with lower membrane GPL signals and higher Lyso-GPL-related signals and could have contributed to FFA accumulation. This interpretation does not require biological upregulation of lipid-hydrolyzing enzymes during HPP; rather, it emphasizes altered substrate–enzyme accessibility as a possible consequence of pressure exposure. Nevertheless, because the annotated lipid coverage was incomplete, GPLs, Lyso-GPLs, and neutral lipids were not absolutely quantified, and lipase, phospholipase, and LOX activities were not directly measured, this structural–enzymatic link remains inferential. Membrane-lipid remodeling is therefore treated as a candidate contributor rather than a resolved primary mechanism, and the present dataset does not establish whether it predominated over neutral acylglycerol hydrolysis or other acyl-release routes.

The unsaturated fatty acid-to-oxylipin/jasmonate map (**Supplementary Figure 3**) provides an additional pathway-level perspective on this response. It highlights coordinated shifts along oxygenation-related nodes, consistent with a potential competing sink that may influence the apparent unsaturated-FFA pattern under combined pressure and hot–humid stress. However, because the current data are not flux-resolved and primary oxidation products were not directly quantified, they cannot partition FFA changes among hydrolytic release, reacylation, oxygenation, and oxidative cleavage. Dedicated turnover measurements, enzyme activity assays, and compartment-resolved lipidomics will be required to determine the relative contribution of these routes. Thus, the present results support a pressure-level-associated reorganization of early lipid handling, but they do not justify assignment of a single dominant biochemical pathway.

### 4.3 Comparison-level metabolomics reveals a shared response backbone and a pressure-amplified expansion component

A common limitation of pairwise metabolomics is that contrast-specific marker lists are often difficult to integrate into a transferable biological interpretation. Here, organizing the untargeted results into shared and treatment-specific discriminants, together with recurrent robust markers across the broader six-comparison framework, helped address this limitation (**Figures 5**-**7**; **Supplementary Tables 2**-**S4**). Under the same hot–humid holding window, the shared discriminants defined a reproducible response backbone, whereas the treatment-specific sets captured regime-specific extensions of that backbone. The substantially larger D-specific set, together with the strongest multivariate displacement under 500 MPa, indicates that pressure primarily amplified the treatment-specific expansion component of divergence rather than merely strengthening a shared response.

At the phenotype interface, however, these untargeted layers did not collapse onto a single lipid axis. Signature-tier scores and robust-marker module scores showed selective coupling to targeted FFA metrics (**Figure 9C**), whereas other relationships remained weak or directionally discordant. This selective convergence argues against an oversimplified view in which all metabolome separation is driven by the same lipid process. Instead, lipid mobilization appears to explain a substantial, but incomplete, fraction of the discriminant organization, with additional regime-dependent dimensions contributing in parallel. Consistent with this interpretation, chemical-superclass enrichment differed across tiers (**Figure 9D**), indicating that regime-associated divergence was also reflected in the chemical composition of the discriminant space. More broadly, the persistent incompleteness of untargeted annotation highlights the value of knowledge-guided, multilayer analytical strategies for constraining biologically plausible interpretations without overstating mechanistic conclusions (He et al., 2022; Zheng et al., 2024).

### 4.4 A sequence-dependent sensitivity window reframes postharvest trade-offs in oil-tea seeds

Postharvest handling of *C. oleifera* seeds is often framed as a matter of completing maturation while controlling moisture and temperature to preserve oil quality, with process trajectories commonly monitored using acid and peroxide values together with lipase- and LOX-linked indices (Yu et al., 2023; Zhu et al., 2020, 2024). Within this seed-level model, our data add a process-order dimension to this framework: a short hot–humid window alone produced only limited lipid mobilization, whereas the same window was converted into a pressure-level-dependent mobilization regime after HPP, most prominently at 500 MPa. Interpreted together with the selective metabolome-to-FFA alignment patterns (**Figure 9**) and the conservative dual-channel lipid evidence, including its audit view (**Figure 10**; **Supplementary Figure 4**), the most defensible implication is that upstream pressure exposure may sensitize the seed lipid system to subsequent warm, high-humidity holding. Because no microstructural imaging was performed, this sensitization should be viewed as an inferred process-level effect rather than direct evidence of pressure-induced tissue structural change.

This interpretation also distinguishes the present HPP response from the thermal or thermal-assisted pretreatments discussed above. In those studies, improved oil release or quality modulation is often evaluated after drying or extraction and may reflect the combined effects of matrix loosening, moisture removal, and partial inactivation of lipid-degrading enzymes (Luo et al., 2025; Wang et al., 2022; Xiong et al., 2025). By contrast, the present study captured seed-level FFA accumulation during a short pre-drying hot–humid interval after pressure exposure. The magnitude of the HPP-induced FFA increase should therefore not be directly compared with oil-level acid value, peroxide value, or extraction-yield outcomes reported for microwave, infrared, or other pretreatments, because the measured endpoints and processing sequences are not equivalent. The more relevant distinction is mechanistic and process-based: thermal pretreatments may combine structural disruption with enzyme attenuation, whereas HPP may primarily alter compartmental constraints and thereby increase the sensitivity of moist oilseeds to acyl release during subsequent hot–humid holding. Side-by-side studies under harmonized pretreatment, holding, drying, and extraction conditions will be needed to determine whether HPP produces a stronger, weaker, or mechanistically distinct lipid response relative to other physical pretreatments.

From a practical perspective, this means that HPP should not be evaluated as an isolated front-end intervention, but rather as one component of a treatment sequence whose compatibility with immediate downstream stabilization will determine its net lipid outcome. Under this logic, rapid drying, cooling, or other prompt stabilization steps would be expected to redirect trajectories in the opposite direction by narrowing the effective window for lipid mobilization (Wang et al., 2023; Xiong et al., 2025). At the same time, quality preservation in camellia oil cannot be reduced to enzyme-linked deterioration alone: non-enzymatic chemistry during heating or drying also contributes to quality evolution (He et al., 2023), and volatile profiles may shift in parallel with lipid-related changes (Li et al., 2022; Zeng et al., 2024; Zhang et al., 2023). Our results complement this literature by identifying an earlier postharvest decision window in which the sequence of pressure exposure and hot–humid holding can shape early lipid outcomes. In this sense, the present study reframes postharvest trade-offs in oil-tea seeds not simply as a question of whether a pretreatment is beneficial or detrimental, but of which process sequence gives rise to which lipid trajectory.

## 5 Conclusions

This study shows that HPP reshaped early lipid mobilization in *C. oleifera* seeds within a controlled seed-level hot–humid postharvest model. Hot–humid holding alone caused only modest changes in total FFA abundance, whereas prior HPP converted the same holding period into a pressure-dependent lipid-mobilization response. This response was strongest at 500 MPa, where targeted GC-MS profiling revealed a marked increase in total FFA burden and an unsaturation-enriched free-acyl pool. Untargeted metabolomics further showed that the 500 MPa regime produced the broadest metabolic divergence, although its correspondence with targeted lipid metrics was selective rather than global.

Together, these findings identify HPP as a pressure-sensitive modifier of early seed lipid trajectories within the tested seed-focused model, rather than as an indicator of improved or impaired oil quality or as a proxy for whole-fruit commercial storage. The semi-quantitative lipid-contextual results are compatible with membrane-lipid remodeling and oxygenation-associated metabolism as candidate contributors, but they do not resolve the dominant biochemical route underlying FFA accumulation. Accordingly, the process-sequence model proposed here should be regarded as a working hypothesis derived from a controlled postharvest system, not as a validated framework for general postharvest management. Future studies should test this model across different fruit lots, moisture states, holding durations, drying strategies, and scale-up conditions, while directly measuring oil-quality indices, lipid oxidation products, microstructural changes, and lipid-hydrolyzing enzyme activities.

## Data Availability

The processed metabolomics dataset supporting this study has been deposited in Mendeley Data (Wu, 2026), and additional data are available from the corresponding author upon reasonable request. This dataset includes the processed metabolomics matrix and metabolite annotation information used in this study, including identification confidence levels, chemical classification, and relative abundance values for all treatment groups and biological replicates. Additional raw data and extended datasets are not publicly released at this time because they form part of ongoing unpublished follow-up studies. Requests to access the datasets should be directed to the corresponding author.
