# Supplementary material for: High-pressure processing reshapes early lipid mobilization in Camellia oleifera seeds during a hot–humid postharvest window
Source: Front Plant Sci. 2026 May 21;17:1829285. doi: 10.3389/fpls.2026.1829285 (PMC13233364; doi:10.3389/fpls.2026.1829285)
Supplement: Supplementary file 1 [file DataSheet1.pdf]

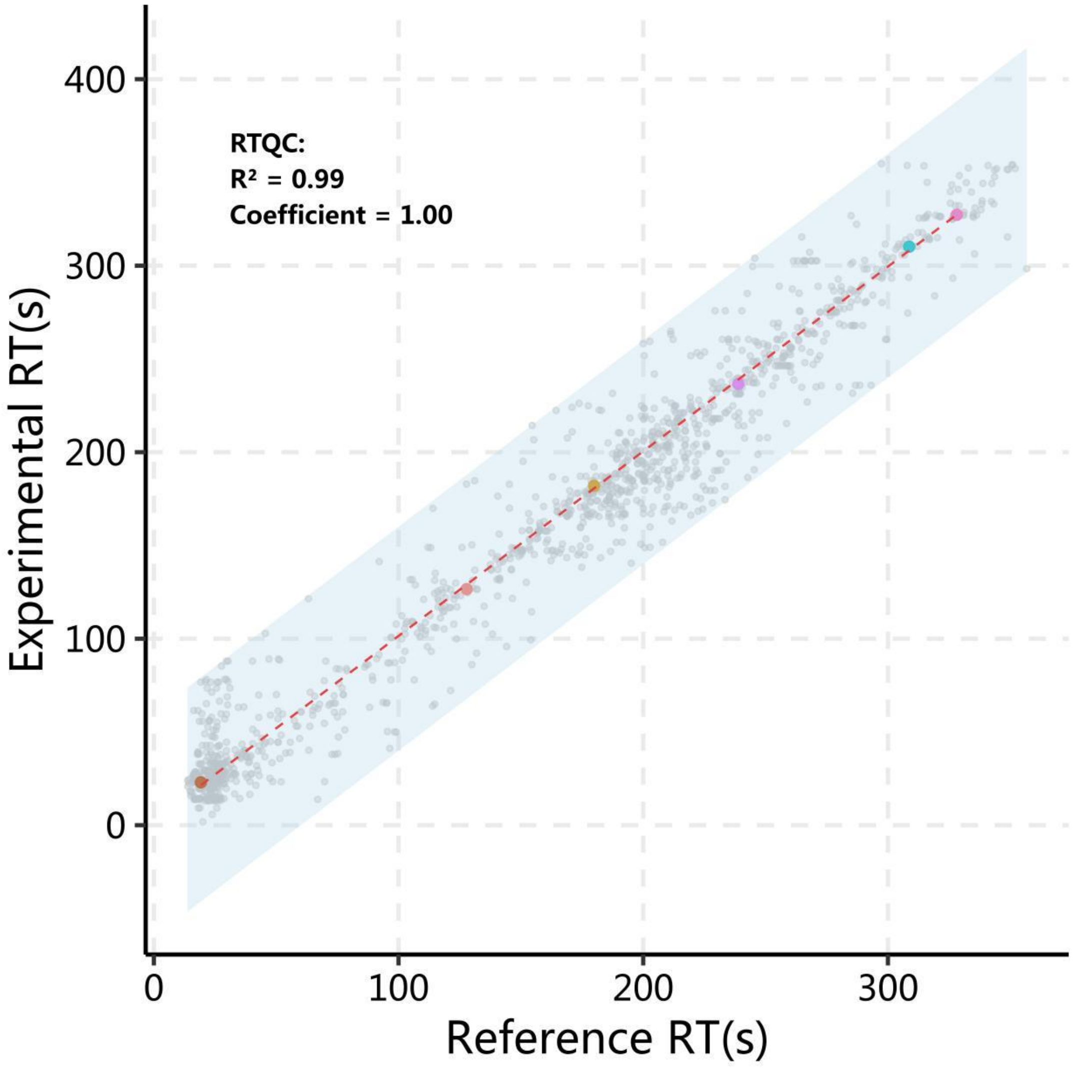

Fig. S1. Retention-time quality-control (RTQC) assessment for the untargeted LC-MS workflow. Scatter plot of reference retention time versus experimental retention time for the RTQC standard mixture across repeated injections. The strong agreement between reference and observed retention times (R^2 = 0.99; slope = 1.00) indicates excellent chromatographic stability and supports robust retention-time alignment across the untargeted LC-MS dataset.
