# Supplementary material for: High-pressure processing reshapes early lipid mobilization in Camellia oleifera seeds during a hot–humid postharvest window
Source: Front Plant Sci. 2026 May 21;17:1829285. doi: 10.3389/fpls.2026.1829285 (PMC13233364; doi:10.3389/fpls.2026.1829285)
Supplement: Supplementary file 2 [file DataSheet2.pdf]

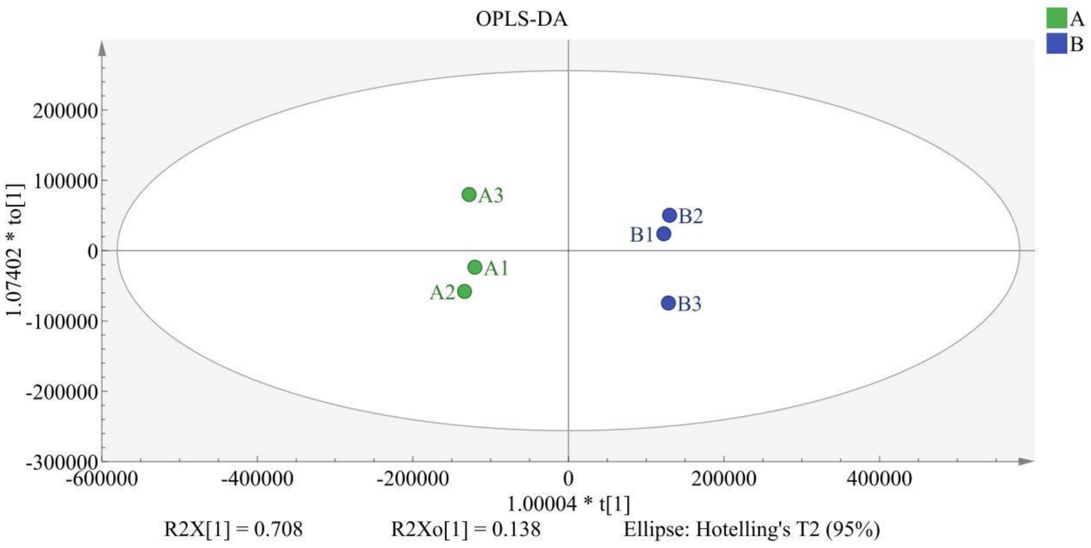

A VS B

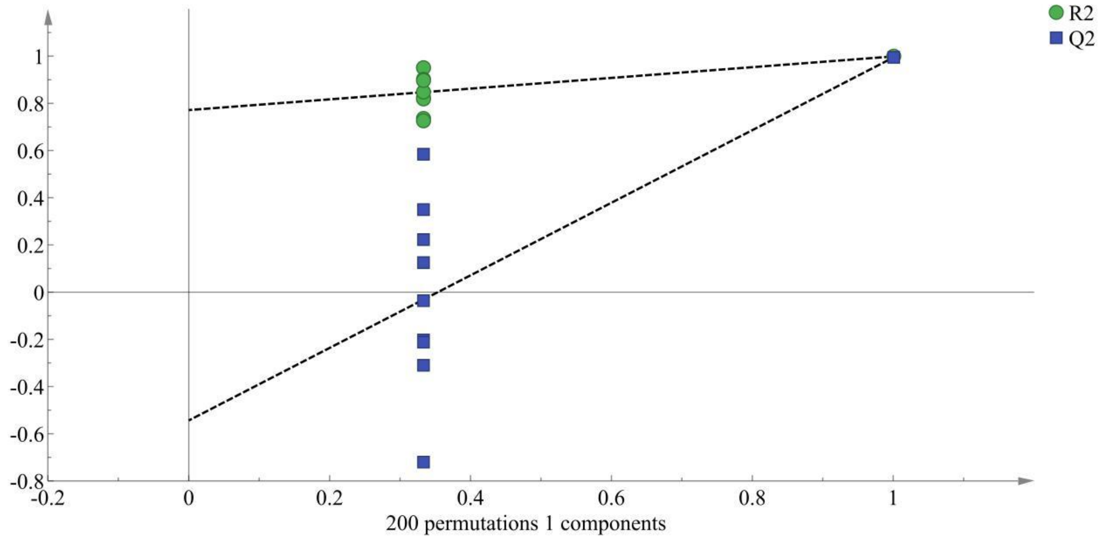

A VS B

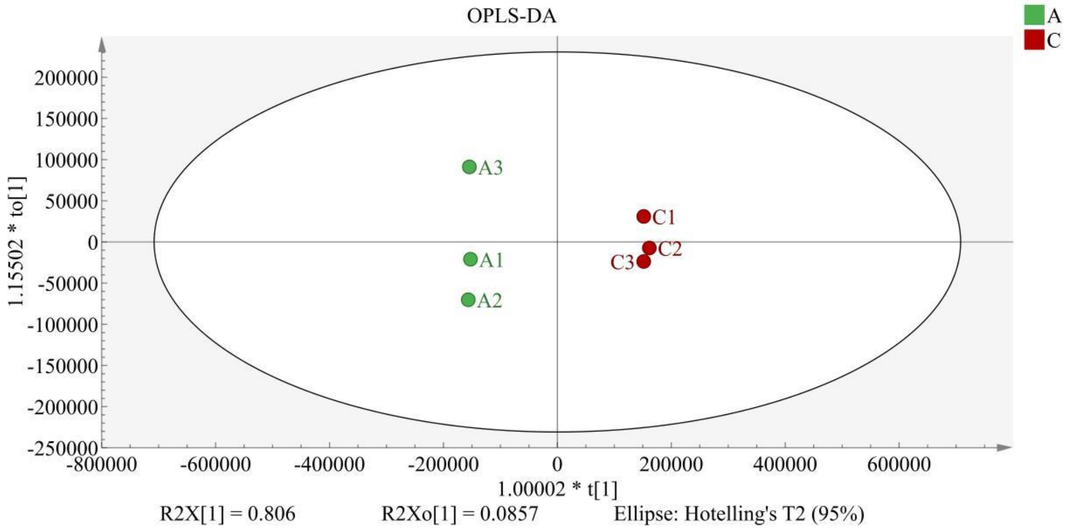

A VS C

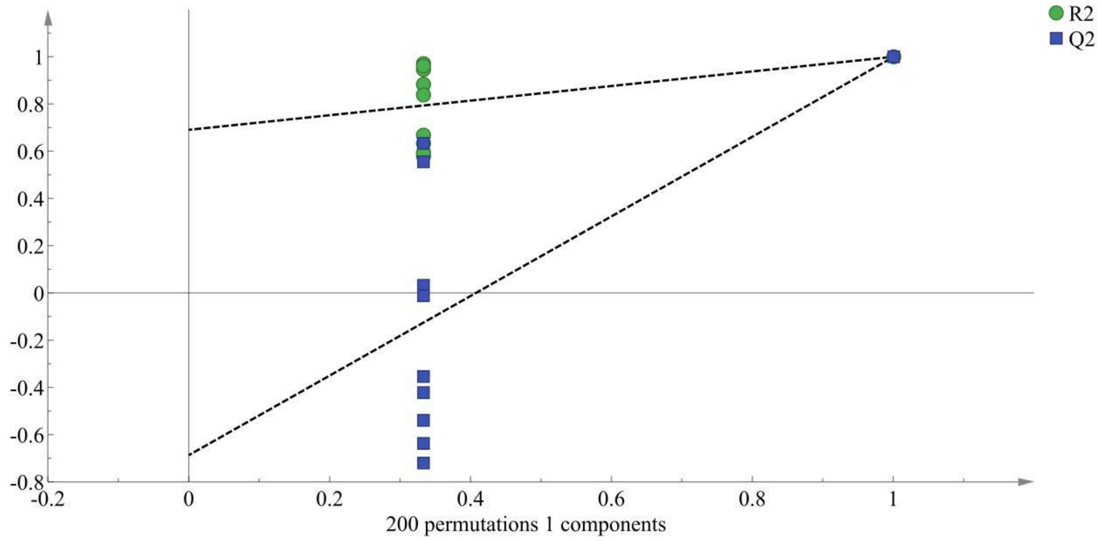

A VS C

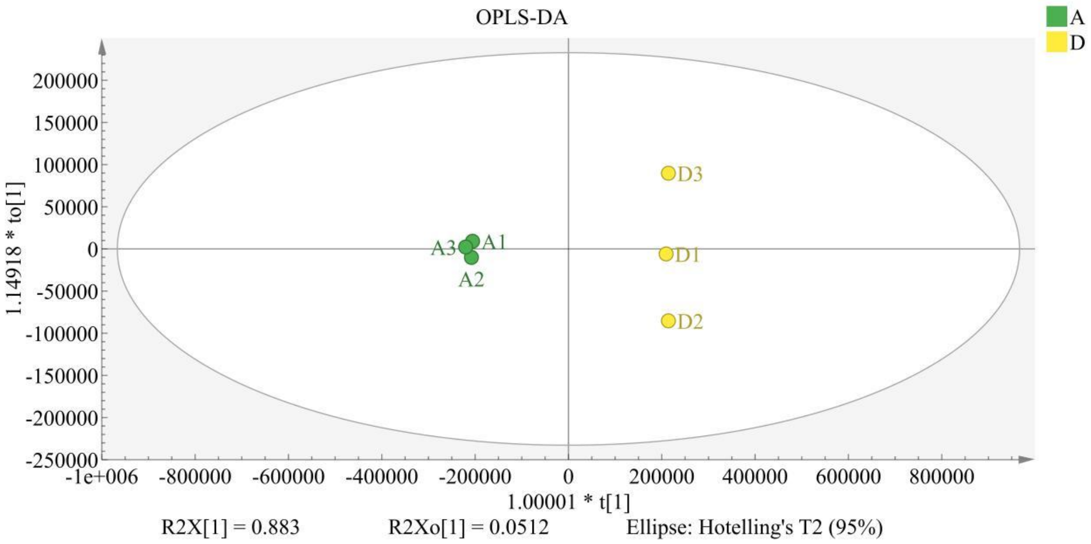

A VS D

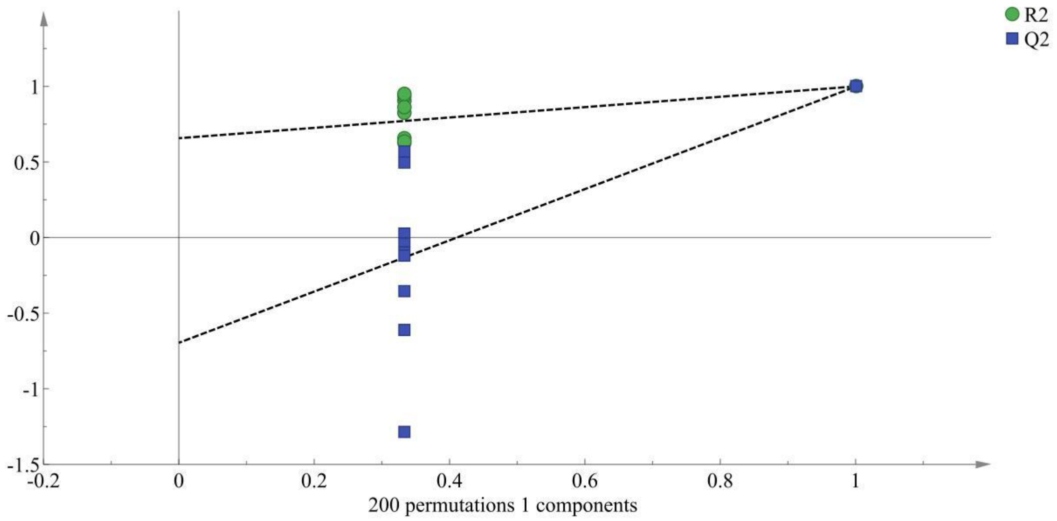

A VS D

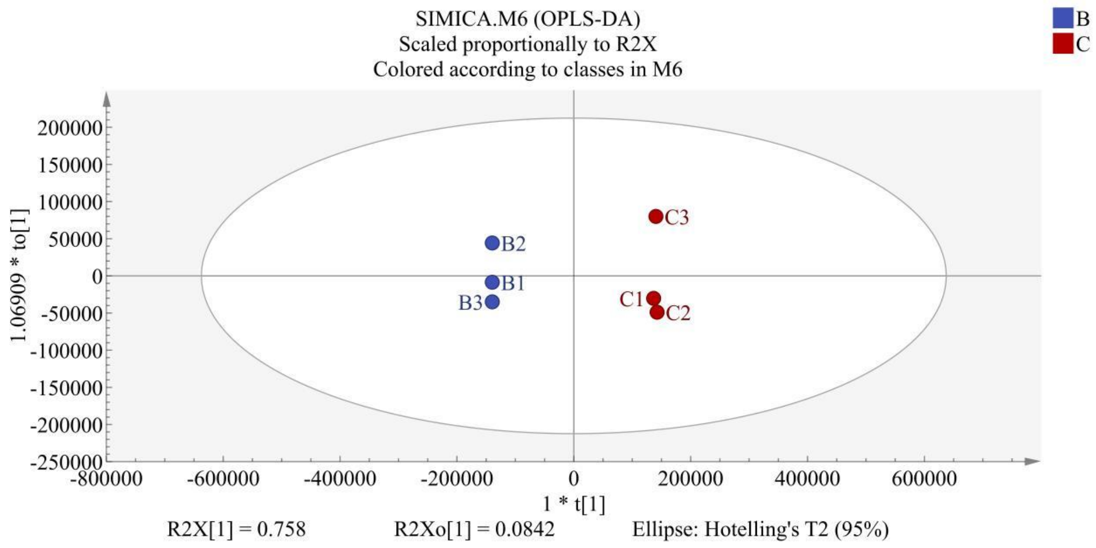

B VS C

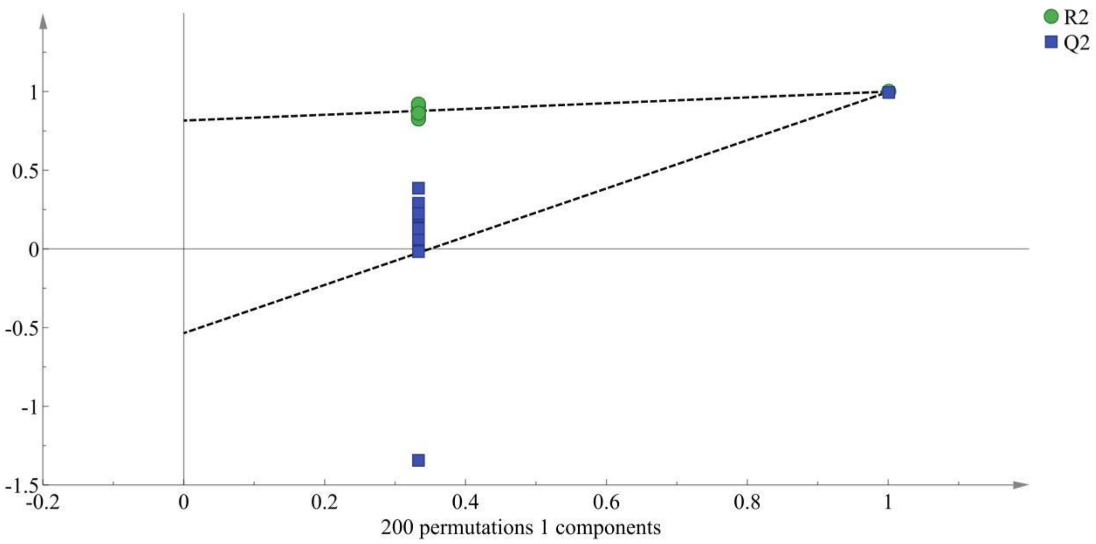

B VS C

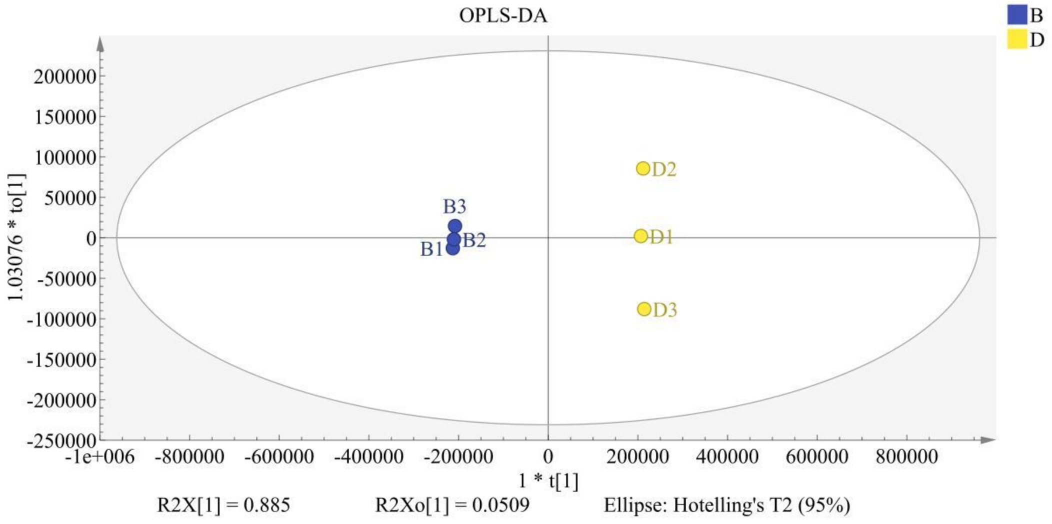

B VS D

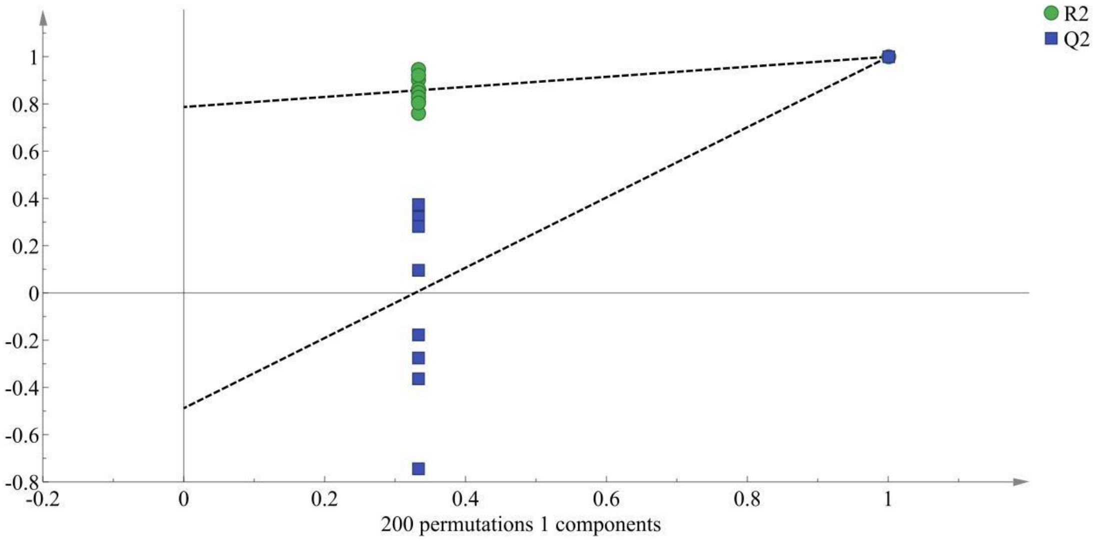

B VS D

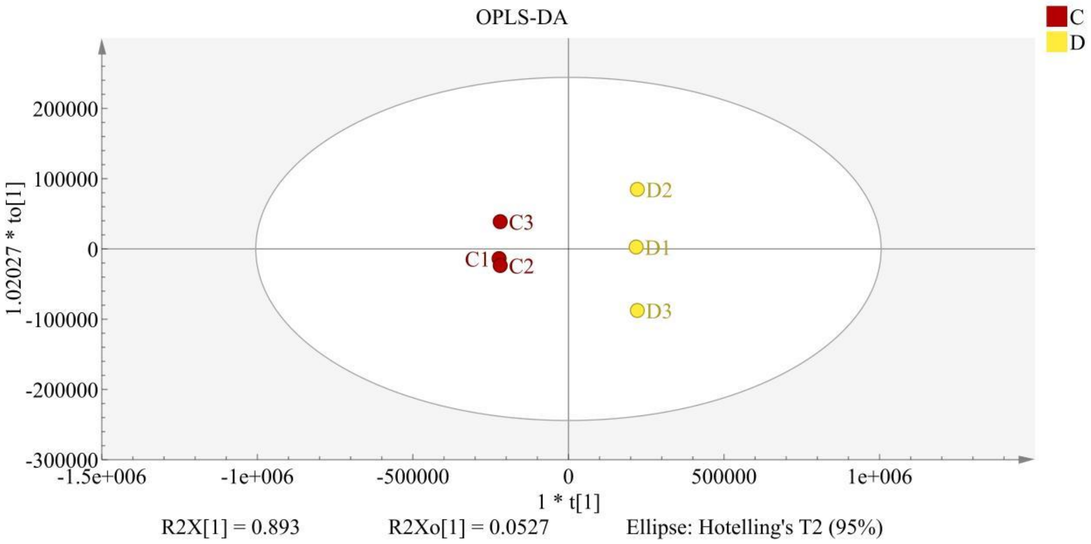

C VS D

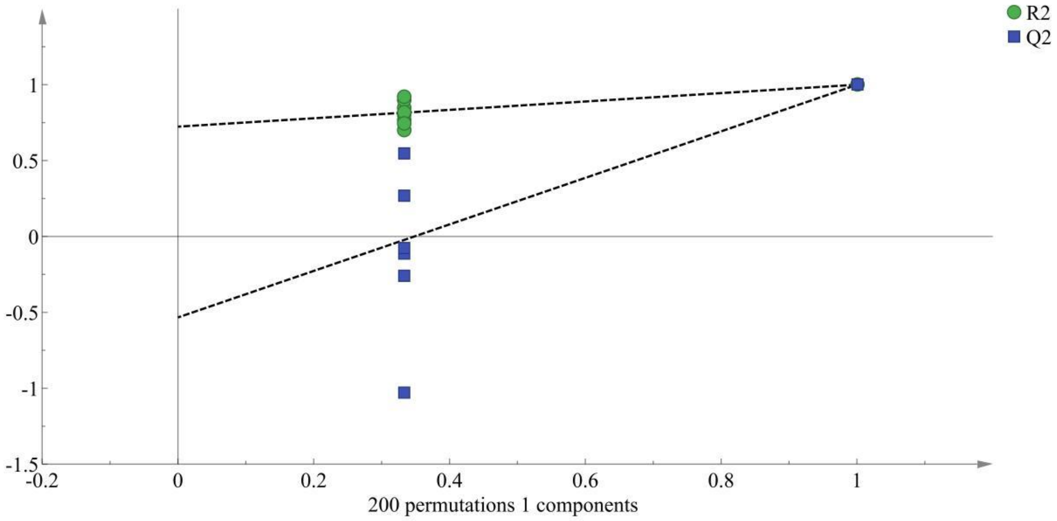

C VS D

Fig. S2. Pairwise OPLS-DA score plots and permutation-based validation across the six regime contrasts.

Pairwise OPLS-DA score plots and the corresponding 200-permutation tests for A vs B, A vs C, A vs D, B vs C, B vs D, and C vs D, based on the MSI Level 1–2 annotated metabolite matrix. The score plots show treatment separation within each contrast, whereas the permutation panels summarize model robustness relative to permuted models. These pairwise models were used to support comparison-level feature prioritization under the prespecified screening criteria and were interpreted as supportive rather than primary evidence.
