# Supplementary material for: High-pressure processing reshapes early lipid mobilization in Camellia oleifera seeds during a hot–humid postharvest window
Source: Front Plant Sci. 2026 May 21;17:1829285. doi: 10.3389/fpls.2026.1829285 (PMC13233364; doi:10.3389/fpls.2026.1829285)
Supplement: Supplementary file 3 [file DataSheet3.pdf]

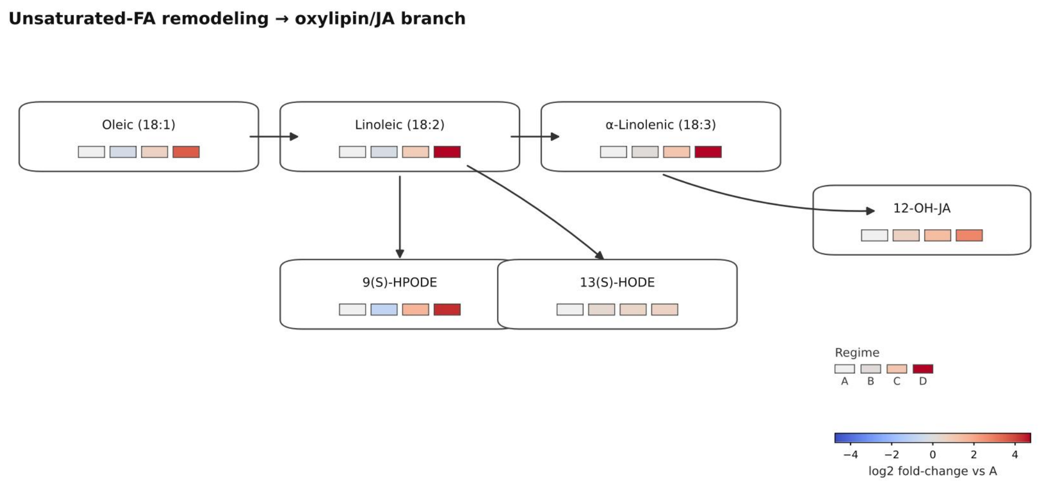

Fig. S3. Regime-dependent shifts in unsaturated acyl pools and oxygenation-related mediators.

Key unsaturated free fatty acids [oleic acid (18:1), linoleic acid (18:2), and α-linolenic acid (18:3)], together with representative oxylipin- and jasmonate-related mediators [9(S)-HPODE, 13(S)-HODE, and 12-OH-JA], were mapped onto a simplified unsaturated-fatty-acid remodeling and oxygenation branch. For each compound, four tiles (left to right) correspond to regimes A–D [A, CK-0 h; B, hot–humid holding only; C, HPP at 100 MPa followed by hot–humid holding; D, HPP at 500 MPa followed by hot–humid holding]. Tile colors represent mean log2 fold-change relative to A (n = 3 biological replicates per regime), using the scale shown. Arrows indicate canonical biochemical relationships and are provided for pathway context only. This map is descriptive and is intended to provide pathway-oriented context for the targeted FFA results rather than direct evidence of flux partitioning.
