# Supplementary material for: High-pressure processing reshapes early lipid mobilization in Camellia oleifera seeds during a hot–humid postharvest window
Source: Front Plant Sci. 2026 May 21;17:1829285. doi: 10.3389/fpls.2026.1829285 (PMC13233364; doi:10.3389/fpls.2026.1829285)
Supplement: Supplementary file 4 [file DataSheet4.pdf]

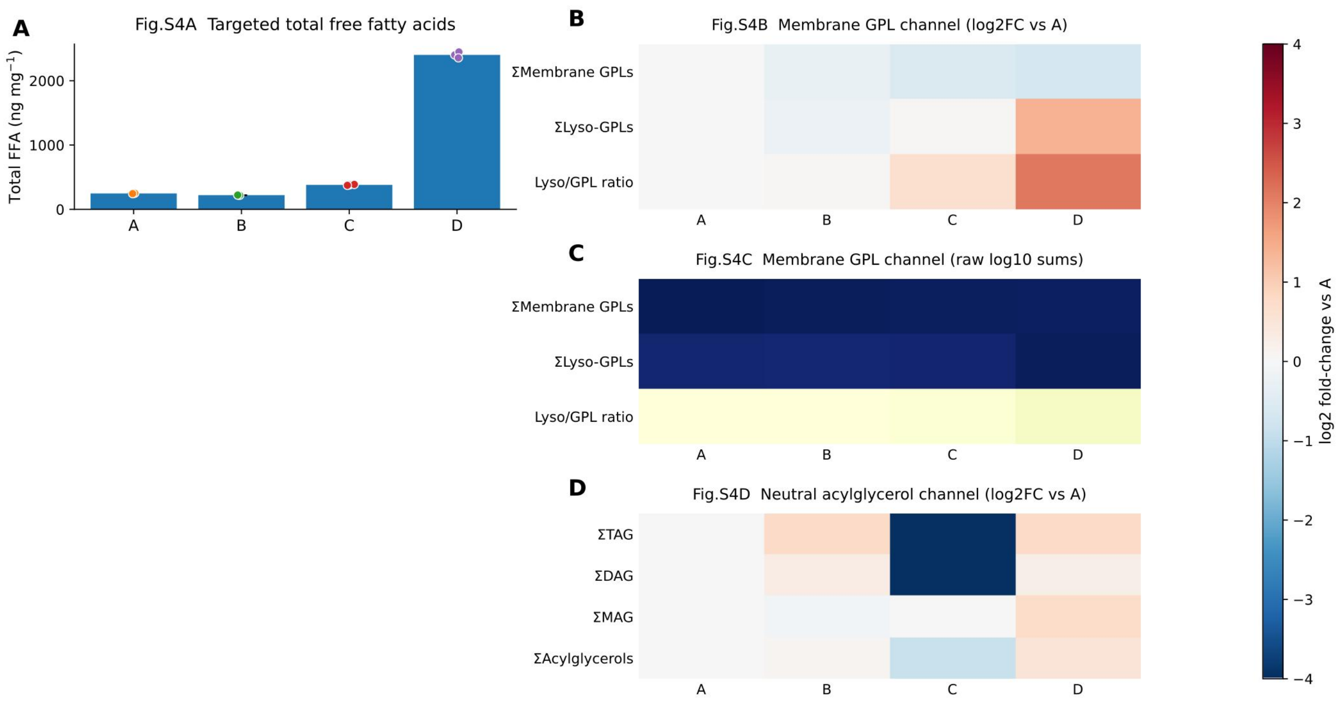

Heatmaps clipped at ±4 for display; raw sums shown as log10.

Fig. S4. Audit view supporting the dual-channel lipid summary shown in Fig. 10.

(A) Targeted total FFAs, as in Fig. 10A. (B) Membrane glycerophospholipid (GPL) channel displayed as log2 fold-changes relative to regime A, as in Fig. 10B. (C) The corresponding raw regime-wise membrane-channel totals shown on a log10 scale to facilitate auditing of directionality independently of fold-change scaling. (D) Neutral acylglycerol channel displayed as log2 fold-changes relative to regime A, as in Fig. 10C. Heatmaps are clipped at ± 4 for display. Raw totals use

pseudocount handling for zero values as described in the source data. These audit panels are derived from relative intensities of confidence-ranked untargeted annotations and should therefore be interpreted as semi-quantitative, regime-aligned evidence rather than absolute lipid quantification. Source data: Table S6.
